# Metabolomics and molecular networking approach for exploring the anti-diabetic activity of medicinal plants†

**DOI:** 10.1039/d3ra04037b

**Published:** 2023-10-19

**Authors:** Arjun Prasad Timilsina, Bimal Kumar Raut, Chen Huo, Karan Khadayat, Prakriti Budhathoki, Mandira Ghimire, Rabin Budhathoki, Niraj Aryal, Ki Hyun Kim, Niranjan Parajuli

**Affiliations:** a Biological Chemistry Lab, Central Department of Chemistry, Tribhuvan University Kirtipur Kathmandu 44618 Nepal niranjan.parajuli@cdc.tu.edu.np +977-1-4332034; b School of Pharmacy, Sungkyunkwan University Suwon 16419 Republic of Korea khkim83@skku.edu +82-31-290-7700; c Department of Biology, University of Florida Gainesville FL 32611 USA

## Abstract

Metabolomics and molecular networking approaches have expanded rapidly in the field of biological sciences and involve the systematic identification, visualization, and high-throughput characterization of bioactive metabolites in natural products using sophisticated mass spectrometry-based techniques. The popularity of natural products in pharmaceutical therapies has been influenced by medicinal plants with a long history of ethnobotany and a vast collection of bioactive compounds. Here, we selected four medicinal plants *Cleistocalyx operculatus*, *Terminalia chebula*, *Ficus lacor*, and *Ficus semicordata*, the biochemical characteristics of which remain unclear owing to the inherent complexity of their plant metabolites. In this study, we aimed to evaluate the potential of these aforementioned plant extracts in inhibiting the enzymatic activity of α-amylase and α-glucosidase, respectively, followed by the annotation of secondary metabolites. The methanol extract of *Ficus semicordata* exhibited the highest α-amylase inhibition with an IC_50_ of 46.8 ± 1.8 μg mL^−1^, whereas the water fraction of *Terminalia chebula* fruits demonstrated the most significant α-glucosidase inhibition with an IC_50_ value of 1.07 ± 0.01 μg mL^−1^. The metabolic profiling of plant extracts was analyzed through Liquid Chromatography-Mass Spectrometry (LC-HRMS) of the active fractions, resulting in the annotation of 32 secondary metabolites. Furthermore, we applied the Global Natural Product Social Molecular Networking (GNPS) platform to evaluate the MS/MS data of *Terminalia chebula* (bark), revealing that there were 205 and 160 individual ion species observed as nodes in the methanol and ethyl acetate fractions, respectively. Twenty-two metabolites were tentatively identified from the network map, of which 11 compounds were unidentified during manual annotation.

## Abstract 2

Metabolomics and molecular networking analysis involve the systematic identification, visualization, and high-throughput characterization of bioactive metabolites in natural products.

## Graphical abstract

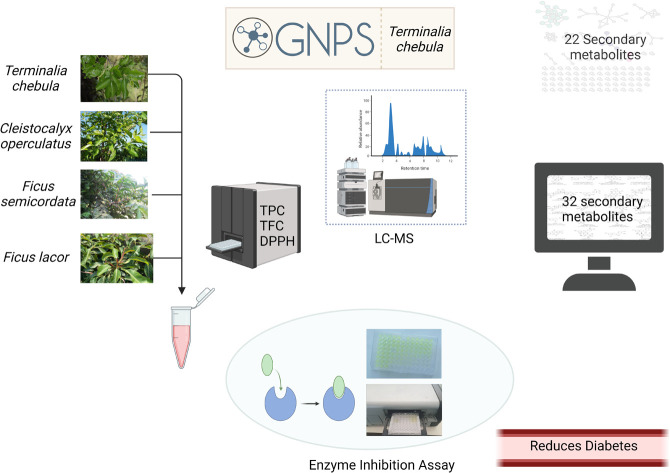

## Introduction

Despite considerable advancements in synthetic chemistry, the search for new and innovative pharmaceuticals is perpetual, and nature continues to be a crucial source of potential new medicines. Recent instances of natural product drug development have highlighted the use of genomic and metabolomics methodologies to supplement conventional methods of natural product research. Medicinal plants contain a range of bioactive metabolites with therapeutic properties. Therefore, robust techniques and technological advancements have been used to explore phytochemistry.

The field of metabolomics has been rapidly growing over the past two decades owing to its ability to provide information on dozens to hundreds of metabolites in a single experiment. It includes the identification and quantification of endogenous and exogenous metabolites present in biological samples using highly sensitive analytical tools and bioinformatics.^1^ Significant advancements in metabolomics have enhanced its applicability to biomarker investigations, toxicological analysis, drug discovery, and natural product chemistry.^2^ The metabolic data acquired in plant metabolomics using single analytical tools are often insufficient and incomplete, which has led the scientific community to utilize MS-based techniques such as GC-MS and LC-MS.^3^ Complex MS/MS data acquired in metabolomics experiments can be visualized and analyzed using a computer-based approach, such as molecular networking. The Global Natural Product Social Molecular Networking (GNPS) is an online bioinformatics platform currently being utilized in research to perform molecular networking. It tends to detect possible similarities among all MS/MS spectra in the dataset and extend annotation to unknown but closely related metabolites.^4,5^

Diabetes is an unassailable burden in low and high-income countries.^6^ Managing diabetes remains a significant challenge for the scientific community since individuals with this condition are vulnerable to various health complications resulting in the annual mortality of 1.5 million people.^7^ Consequently, promoting research on medicinal plants for the identification of new alternative therapeutics for diabetes is currently relevant due to favorable outcomes associated with plant-based therapeutics in terms of long-term safety, mode of action, and metabolic activity.

Based on their ethnobotanical pharmaceutical properties, four medicinal plants, namely *C. operculatus*, *T. chebula*, *F. lacor*, and *F. semicordata*, were selected in this study. Although these plants have a wide range of pharmacological applications, this study intended to investigate the applicability of these species and their related metabolites as anti-diabetic drugs. The efficacy and toxicity of herbal formulations must be thoroughly explored even though they are prepared and consumed locally. Finding viable drug candidates from crude extracts is more challenging due to the complexity of biological samples.^8^

Various classes of drugs like insulin, α-glucosidase inhibitors (acarbose, miglitol, voglibose), biguanides (metformin, phenformin), thiazolidinediones, sulfonylureas, dipeptidyl-peptidase-4 inhibitors, meglitinides, dopamine agonists have currently being used to treat diabetes. However, these are reported to have several side effects such as allergic reactions, increased risk of bladder cancer, hypoglycemia, bloating, flatulence, *etc.*^9^ As a result, the issue demands the development of new potent drugs with no or minimal human side effects.

The purpose of this research was to explore secondary metabolites using a mass spectrometry-based metabolomics approach with the GNPS platform and to evaluate the possible enzyme inhibitory activity of *C. operculatus*, *T. chebula*, *F. semicordata*, and *F. lacor* in targeting diabetes and its complications.

## Materials and methods

### Chemicals

Methanol, ethanol, ethyl acetate, dichloromethane, and hexane were purchased from Thermo Fisher Scientific (India). Gallic acid and quercetin were procured from HiMedia, an Indian supplier. α-Glucosidase (*Saccharomyces cerevisiae*), 4-nitrophenyl-α-d-glucopyranoside (pNPG), pancreatic porcine α-amylase (PPA), 2-chloro-4-nitrophenyl-α-d-maltotrioside (CNPG3), and acarbose were ordered from Sigma-Aldrich (Germany).

### Medicinal plants

Medicinal plants were collected from four distinct locations in Nepal, and the herbarium of each plant was submitted to the Central Herbarium of the Central Botany Department at Tribhuvan University, where voucher specimens were subsequently registered (Table 1). The entire collection of plant samples was shade-dried and pulverized into powder.

**Table 1:** Medicinal plants used in the study and their ethnobotanical uses and reported chemical constituents

Voucher specimen	Medicinal plant	Family	Ethnobotanical uses	Chemical constituents
TUCH 210018	*Terminalia chebula*	Combretaceae	It is used for antidiabetic, anticancer, cardioprotective, neuroprotective, anti-inflammatory, and antiarthritic activities.^10^ It is also used to treat dyspepsia, piles, hepatosplenomegaly, irritable bowel syndrome, and heart failure.^11^	Gallic acid, digallic acid, ellagic acid, caffeic acid, pyragallol, rutin, quercetin, isoquercetin, chebulagic acid, chebulinic acid, chebulic acid, eugenol, terflavin A, terchebulin^10^
TUCH 210017	*Cleistocalyx operculatus*	Myrtaceae	It is used for its cytotoxic,^12^ antitumor,^13^ antihyperglycemic,^14^ cardiotonic,^15^ and anti-inflammatory^16^ activities	2′,4′-Dihydroxy-6′-methoxy-3′,5′-dimethylchalcone and ursolic acid, 5,7,8,4′-tetrahydroxy-3′-5′-dimethoxyflavone-3-*O*-β-d-galactopyranoside, gossypetin-8, 3′-dimethyl ether-3-*O*-β-d-galactoside, myricetin-3′-methyl ether-3-*O*-β-d-galactopyranoside, myricetin-3′-methyl ether, quercetin, kaempferol^16^
TUCH 201022	*Ficus lacor*	Moraceae	The powdered ripe fruit is utilized as an anti-diabetic.^17^ It is also used for wound healing^18^ ulcers,^19^ hay fever, dysentery, and stomach disorder.^18–21^	β-Sitosterol, lupeol, α-amyrin, β-amyrin, stigmasterol, campesterol, scutellarein glucoside, scutellarein, infection, sorbifolin, bergaptol, and bergapten^22,23^
TUCH 201020	*Ficus semicordata*	Moraceae	For headaches and diarrhea, raw fruit juice and fruits are employed. Fruits are also used for visceral ulcers, jaundice, colic pain, and hepatitis. Externally, the juice of the leaves is useful to treat scabies. It has been shown to have antioxidant, antibacterial, and anticancer properties.^24^	Catechin, rutin, quercetin, dodecane, indole, linalool, gallic acid and germacrene^25^

### Methanolic extracts and fractionation

Methanolic extracts of four plants (*Cleistocalyx operculatus*, *Terminalia chebula*, *Ficus lacor*, and *Ficus semicordata*) were prepared using the cold percolation method, which involves the absorption of the powder for a day in methanol before percolation. The same procedure was repeated continuously for 3 days, and the collected solvent was dried at temperatures below 50 °C in the rotary evaporator. After 50 g of methanolic extracts were dissolved in distilled water for the fractionation process, the extracts were separated using the following solvents: hexane, DCM, and ethyl acetate in order of polarity to obtain four fractions from each plant. This procedure was performed three times for each solvent.^26^

### Determination of polyphenolic and flavonoid contents

Total polyphenol and flavonoid contents (TPC and TFC) were quantified using standard Folin–Ciocalteu and aluminum trichloride methods.^27–29^ Both reactions were performed in 200 μL final volume, and absorbance was measured using a microplate reader (SynergyLX, BioTek, Instruments, Inc., USA). Calibration curves were generated by using various concentrations of gallic acid and quercetin. The extract concentrations were calculated and expressed as gallic acid and quercetin equivalents (mg GAE per g and mg QE per g, respectively).

### Antioxidant assay

The antioxidant assay was performed as described previously in literature.^30^ The reaction was performed using an equal volume of plant extracts with 0.1 mM DPPH to maintain a final volume of 200 μL. After 15 min of incubation at room temperature in the dark, the absorbance was recorded at 517 nm. The percentage scavenging activity was determined using the following formula:
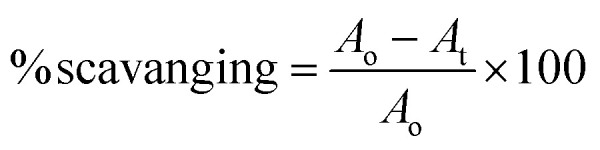
where *A*_o_ = optical density of the negative control (50% DMSO), and *A*_t_ = optical density of the test or reference sample.

### Assay of α-glucosidase inhibition

The α-glucosidase inhibition was performed by dissolving the enzyme and substrate in a 50 mM phosphate buffer (pH 6.8). The process was initiated by mixing 20 μL of plant extracts of varying concentrations with 20 μL enzyme (0.2 units) along with 120 μL phosphate buffer followed by 10 min incubation at 37 °C. After incubation, pNPG substrate was added (0.7 mM) and incubated for 15 min at the same temperature.^31^ The absorbance of the released *p*-nitrophenyl was measured at 405 nm using Synergy LX (BioTek Instruments, Inc., USA). Each test was performed in triplicate. The following formula was used to calculate the % α-glucosidase inhibitory activity
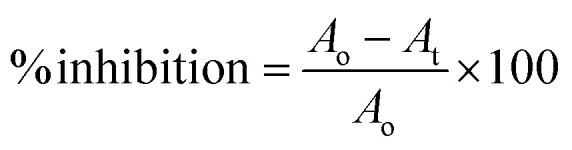
where *A*_o_ is the optical density of the negative control with 30% DMSO and *A*_t_ is the optical density of the test/positive control.

### Assay of α-amylase inhibition

The reaction was performed by dissolving the enzyme and substrate in 50 mM phosphate-buffered saline (pH; 0.9% NaCl, pH 7.0). The assay was inducted by mixing 20 μL plant extracts at different concentrations and adding 80 μL PPA (1.5 units per mL) followed by 10 min incubation at 37 °C. After incubation, CNPG3 (0.5 mM) was added and the cells were incubated for 15 min.^26^ The absorbance was measured at 405 nm using a microplate reader (Synergy LX, BioTek Instruments, Inc., USA). The percentage of inhibition was determined using the following formula:
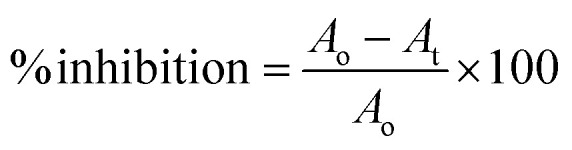
where *A*_o_ is the optical density of the negative control with 30% DMSO and *A*_t_ is the optical density of the test/positive control.

### Liquid chromatography-mass spectrometry analysis

LC-HRMS of the ethyl acetate and water fractions of *T. chebula* fruits, *C. operculatus*, and *F. lacor*, *F. semicordata* were analyzed at the Sophisticated Analytical Instrument Facility (SAIF), CSIR-Central Drug Research Institute, Lucknow (Lot No. 1607642300 and 1606747677). Compounds were identified using an Agilent 1200 series HPLC system (Agilent Technologies, Santa Clara, CA, USA) equipped with an Agilent 6520 I Accurate-Mass Q-TOF LC/MS system (Agilent Technologies, Santa Clara, CA, USA). An Agilent 6520 Accurate-Mass Q-TOF Mass Spectrometer equipped with a G1311A quaternary pump, G1329A autosampler, and G1315D diode array detector (DAD) was used for further analysis. The elution solvent comprised a mixture of acetonitrile, 5 mM acetate buffer, and water flowing at 1.5 mL min^−1^, following a gradient from 5% to 30% acetonitrile over 10 minutes, reaching 80% acetonitrile over 32 minutes, and returning to the initial conditions, with the column temperature consistently held at 30 °C throughout the process. For positive mode analysis of electrospray ionization, the source gas temperature was maintained at 30 °C, with a gas flow rate of 11.01 L min^−1^, and a nebulizer pressure of 40 psi. VCap voltage was set to 3500 V, fragmentor at 175 V, skimmer at 65.0 V, and Octapole RF peak at 750 V. The MS data acquisition was done in the range of 100–2000 Da at a scan rate of 1.03 for *T. chebula* (fruit), *C. operculatus*, *F. lacor,* and *F. semichordata*.^32^ Moreover, the molecular annotation of the methanolic extract (AT1) and ethyl acetate fraction (AT2) of *T. chebula* bark was completed in SIRIUS 5.6.3, using the CSI: FingerID interface, where the efficiency of the predicted molecular formula, functional group, and fragmentation pattern was evaluated in terms of the Sirius score.^33^

### HRMS and GNPS-based metabolomic analysis

Samples of AT1 and AT2 were prepared for MS/MS analysis by dissolving them in HPLC-grade (1 mg mL^−1^), and then 150 μL volume of each sample was transferred to an HPLC autosampler vial. Metabolomic profiling of the two samples was performed using an Agilent G6545B quadrupole time-of-flight (Q-TOF) mass spectrometer (Agilent Technologies, Santa Clara, CA, USA) equipped with a heated electrospray ion source (HESI). For chromatographic separation, an Acquity® UPLC BEH reverse-phase column C18 (150 mm × 2.1 mm, 1.7 μM) was used. The mobile phase was acidified with 0.1% formic acid and comprised of H_2_O (A) and acetonitrile (B). The composition of organic solvent was 5% at 0.00–2.00 min, 20% at 5.00 min, 100% at 20.00 min, and back again to 5% at 23.00–25.00 min. The injection volume of two samples was kept at 3 μL and a flow rate of 0.5 mL min^−1^ was maintained. MS/MS analysis was performed using electrospray ionization (ESI) in positive ion mode. Spectral hits were performed using a modified version of the method reported by Bashir *et al.* (2021)^34^ with an *m*/*z* range of 50–1200, collision energies of 15 eV and 40 eV, and a full width at half maximum (FWHM) of 3000. The RAW files were converted to open-source ‘.mzXML’ file format using the MSConvert tool of ProteoWizard MSConvert Version 3 software. The files were uploaded to the GNPS platform using WinSCP, the recommended FTP client. The acquired MS^2^ data were visualized using a GNPS-based visualization following an established procedure (accessed on January 17, 2023). The molecular networks generated were further exported from GNPS to Cytoscape in ‘.graphml’ format to enable customized visualization.

### Data analysis

The collected data were processed using Gen5 Microplate Software, followed by MS Excel. GraphPad Prism Software version 8 was used for IC_50_ calculation and data were represented as a mean ± standard error of the mean of triplicate. The Mnova software ver. 12.0 (Mestrelab Research, Santiago de Compostela, Spain) was used to process the raw data files obtained from the HPLC-QTOF-MS.

## Results

### Estimation of polyphenol contents and antioxidant potential

Phytochemical and antioxidant assays of the medicinal plants were performed in triplicate using methanolic extracts. The total phenol, flavonoid, and antioxidant activities of the plants are shown in Table 2. *T. chebula* (Bark and Fruit) showed the highest TPC (164.534 ± 3.70 mg GAE per g) and TFC values (158.67 ± 14.68 mg QE per g), respectively. Similarly, *T. chebula* (bark) showed the lowest IC_50_ value of 5.144 ± 0.06 μg mL^−1^ for DPPH radical scavenging activity as compared with the control candidate, quercetin (6.3 ± 1.0 μg mL^−1^).

**Table 2:** Phytoconstituents and antioxidant activity of the methanol extracts of medicinal plants

Sample	TPC (mg GAE per g)	TFC (mg QE per g)	Antioxidant IC_50_ values (μg mL^−1^)
*Terminalia chebula* (fruit)	161.56 ± 0.39	158.67 ± 14.68	34.82 ± 1.12
*Terminalia chebula* (bark)	164.53 ± 3.70	115.34 ± 4.07	5.144 ± 0.06
*Cleistocalyx operculatus*	154.26 ± 1.01	69.17 ± 0.73	30.34 ± 1.12
*Ficus lacor*	108.67 ± 6.37	131.16 ± 7.75	130 ± 1.02
*Ficus semicordata*	107.76 ± 3.21	77.18 ± 0.73	22.76 ± 0.19
Quercetin (control)	—	—	6.3 ± 1.00

### Inhibition of diabetic targets α-amylase and α-glucosidase

Preliminary screening was performed using 500 μg mL^−1^ of the extracts to determine the percentage inhibition of α-glucosidase and α-amylase, respectively. Only fractions with more than 50% inhibition were used to determine the IC_50_ values. As compared to the positive control, acarbose (6.1 ± 0.10 μg mL^−1^), and the lowest IC_50_ value towards α-amylase inhibition was found for *F. semicordata* in methanolic extract (46.8 ± 1.8 μg mL^−1^). Similarly, the lowest IC_50_ value for α-glucosidase inhibition was observed for *T. chebula* (fruits) in aqueous fraction (1.07 ± 0.01 μg mL^−1^) and for *C. operculatus* in methanolic extract (1.26 ± 0.03 μg mL^−1^). The results of α-glucosidase and α-amylase inhibition by different plant fractions are shown in Table 3.

**Table 3:** α-Amylase (A) and α-glucosidase (G) activities of various solvent fractions from medicinal plants^a^

Medicinal plants	Minimum inhibitory concentration, IC_50_ (μg mL^−1^)									
	Methanol ext.		Hexane fr.		Dichloromethane fr.		Ethyl acetate fr.		Water fr.	
	A	G	A	G	A	G	A	G	A	G
*Terminalia chebula* (fruit)	153.2 ± 1.91	95.37 ± 0.30	<50%	236.06 ± 4.78	<50%	<50%	95.5 ± 1.5	12.72 ± 0.40	309.1 ± 0.9	1.07 ± 0.01
*Terminalia chebula* (bark)	180.4 ± 10.22	8.251 ± 1.08	167.5 ± 9.47	<50%	141.2 ± 1.43	<50%	140.6 ± 2.61	6.633 ± 1.82	219.2 ± 3.26	31.96 ± 0.74
*Cleistocalyx operculatus*	62.49 ± 1.89	1.26 ± 0.03	414.9 ± 7.8	80.31 ± 1.22	429.4 ± 5.2	95.19 ± 0.75	100.8 ± 1.9	8.67 ± 0.55	53.2 ± 1.0	4.91 ± 0.02
*Ficus lacor*	485.6 ± 1.8	33.78 ± 3.6	<50%	76.57 ± 0.5	355.2 ± 11.2	<50%	240.1 ± 3.4	94.2 ± 8.1	<50%	76.6 ± 9.7
*Ficus semicordata*	46.8 ± 1.8	46.3 ± 3.07	<50%	<50%	<50%	<50%	241.7 ± 4.3	34.7 ± 1.1	<50%	5.0 ± 0.2
Acarbose	6.1 ± 0.10 (α-amylase)				344.23 ± 1.03 (α-glucosidase)					

a Extract: ext. Fraction: fr.

### Liquid chromatography and molecular annotation

**Fig. 1:**
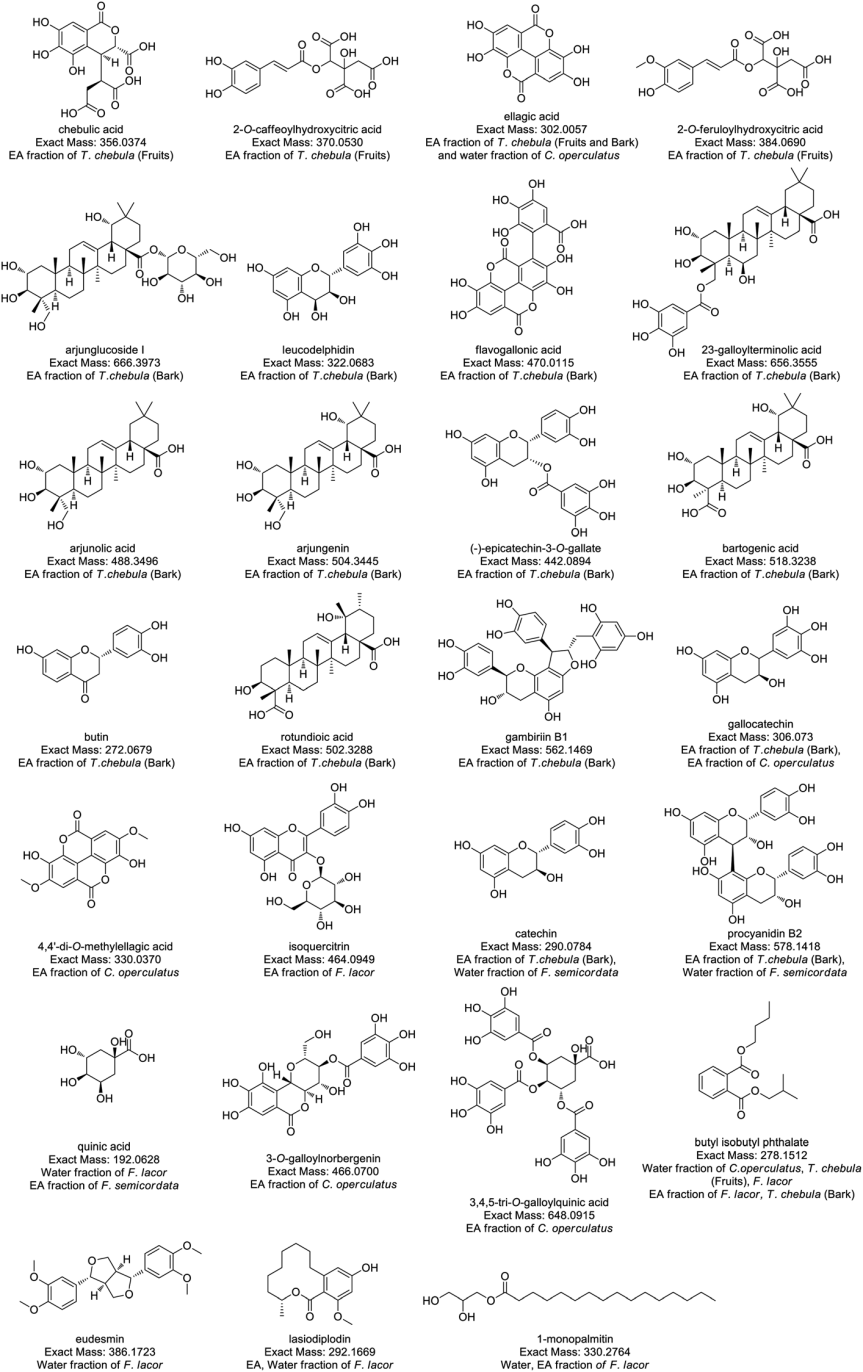
Structure of annotated compounds from *T. chebula*, *F. lacor*, *C. operculatus*, and *F. semicordata* based on LC-HRMS.

Compound identification and characterization were based on the comparison of molecular masses, retention times, and MS spectra *via* authentic databases (PubChem, and Dictionary of Natural Products), as well as relevant literature. Because of the potent anti-diabetic activity exhibited by the ethyl acetate and water fractions of the four plants, the ethyl acetate and water fractions were selected for further molecular annotation analysis. Thirty-two compounds were identified from the ethyl acetate and water fraction of plant species, and the mass spectra of these compounds showed peaks at *m*/*z* 357.0455 (ESI Fig. S1†), 371.0619 (Fig. S2), 303.0140 (Fig. S3), 619.0970 (Fig. S4), 385.0788 (Fig. S5), 667.4054 (Fig. S6), 657.3627 (Fig. S7), 471.0197 (Fig. S8), 323.0762 (Fig. S9), 489.3570 (Fig. S10), 519.3313 (Fig. S11), 505.3529 (Fig. S12), 443.0978 (Fig. S13), 563.1549 (Fig. S14), 503.3363 (Fig. S15), 273.0759 (Fig. S16), 307.0815 (Fig. S17), 331.0463 (Fig. S18), 465.1023 (Fig. S19), 291.0859 (Fig. S20), 579.1504 (Fig. S21), 435.0578 (Fig. S22), 193.0705 (Fig. S23), 487.22 (Fig. S24), 467.0872 (Fig. S25), 649.1082 (Fig. S26), 393.0435 (Fig. S27), 279.1601 (Fig. S28), 387.1817 (Fig. S29), 293.1736 (Fig. S30), 331.2842 (Fig. S31), and 463.3794 (Fig. S32†) as protonated molecular ions [M + H]^+^ as shown in Table 4. In ESI figures, ESI (+) indicates data collected in the positive mode of electrospray ionization mass spectrometry, centroid TIC and centroid MS indicate the data are presented in centroid mode only where in centroid mode each ion's mass and intensity are presented as discrete values, while profile mode indicates the entire distribution of intensity of ions recorded at each *m*/*z* value. Therefore, centroid MS data offers simpler spectra and helps in the interpretation of spectra. The annotated compounds and their fragment's peak were compared with previous findings as listed in Table 4. The structures of annotated molecules were drawn by using http://www.chemspider.com/. The structures of the annotated secondary metabolites are shown in Fig. 1.

**Table 4:** Details of the annotated compounds from ethyl acetate and water fractions^a^

Purposed compound	Calculated mass	Observed mass	Formula	RDBE	Absolute error (ppm)	(Rt) Min	Fragment peak	Solvent fraction and plant species	CSI: Finger ID (score%)
Chebulic acid	356.0374	357.0455	C_14_H_12_O_11_	9	0.77	2.84	339, 321, 293, 203	EA *T. chebula* (fruits)	—
2-*O*-Caffeoyl hydroxy citric acid	370.0530	371.0619	C_15_H_14_O_11_	9	2.88	2.93	191, 163, 145	EA *T. chebula* (fruits)	—
Ellagic acid	302.0057	303.0140	C_14_H_6_O_8_	12	1.52	8.98	193	EA *T. chebula* (fruits)	—
	302.0057	303.0143	C_14_H_6_O_8_	12	2.60	8.70	—	Water *C. operculatus*	—
	302.0057	303.0134	C_14_H_6_O_8_	12	0.31	8.62	201.01, 229.01, 247.02, 201.01, 173.02, 145.02	*T. chebula* (bark)	98.05
Trigalloyllevoglucosan IX	618.0910	619.0970	C_20_H_26_O_22_	8	2.83	10.62	153	EA *T. chebula* (fruits)	—
2-*O*-Feruloylhydroxycitric acid	384.0690	385.0788	C_16_H_16_O_11_	9	4.39	4.58	385, 209, 195	EA *T. chebula* (fruits)	—
Arjunglucoside I	666.3973	667.4054	C_36_H_58_O_11_	8	0.46	10.06	527.33, 483.34	*T. chebula* (bark)	99.41
23-Galloylterminolic acid	656.3555	657.3627	C_37_H_52_O_10_	12	0.94	10.33	639.35, 469.32, 451.31, 405.31, 187.14	*T. chebula* (bark)	55.46
Flavogallonic acid	470.0115	471.0197	C_21_H_10_O_13_	17	0.73	5.68	453.09, 379.00, 363.01	*T. chebula* (bark)	72.99
Leucodelphidin	322.0683	323.0762	C_15_H_14_O_8_	9	0.24	6.85	305.06, 139.03, 217.04, 263.05, 163.03	*T. chebula* (bark)	54.41
Arjunolic acid	488.3496	489.3570	C_30_H_48_O_5_	7	0.92	11.50	471.34, 453.33, 205.15, 135.04	*T. chebula* (bark)	92.54
Bartogenic acid	518.3238	519.3313	C_30_H_46_O_7_	8	0.47	11.18	501.32, 215.14, 119.08, 187.14, 437.30	*T. chebula* (bark)	90.33
Arjungenin	504.3445	505.3529	C_30_H_48_O_6_	7	1.10	10.06	469.33, 451.32, 297.17, 187.14, 173.13, 405.31, 423.32	*T. chebula* (bark)	95.0
(−)-Epicatechin-3-*O*-gallate	442.0894	443.0978	C_22_H_18_O_10_	14	1.41	8.95	139.03, 123.04, 153.01, 273.07, 11.04	*T. chebula* (bark)	99.31
Gambiriin B1	562.1469	563.1549	C_30_H_26_O_11_	18	0.29	9.02	437.12, 411.10, 139.03, 123.04, 273.07	*T. chebula* (bark)	93.01
Rotundioic acid	502.3288	503.3363	C_30_H_46_O_6_	8	0.77	13.40	449.30, 421.31, 485.32, 173.13, 119.08, 95.08, 201.16	*T. chebula* (bark)	82.97
Butin	272.0679	273.0759	C_12_H_22_O_4_	10	0.77	8.95	123.04, 77.03, 51.02	*T. chebula* (bark)	83.65
Gallocatechin or epigallocatechin	306.0734	307.0815	C_15_H_14_O_7_	9	0.89	7.22	289, 307, 139, 151	EA *C. operculatus*	—
	306.0734	307.0814	C_15_H_14_O_7_	9	0.87	6.66	289.07, 139.03, 163.03, 181.04, 65.03	*T. chebula* (bark)	100
4,4′-Di-*O*-methylellagic acid	330.0370	331.0463	C_16_H_10_O_8_	12	4.61	17.48	225, 245, 270, 299, 300.9, 316	EA *C. operculatus*	—
Isoquercitrin	464.0949	465.1023	C_21_H_20_O_12_	12	3.31	8.80	303.04, 137.05	EA *F. lacor*	—
Catechin	290.0784	291.0859	C_15_H_14_O_6_	9	4.30	11.56	123, 139, 165, 207	Water *F. semicordata*	—
	290.0784	291.0866	C_15_H_14_O_6_	9	0.29	7.77	139.03, 123.04, 207.06, 91.05, 95.05	*T. chebula* (bark)	100
Procyanidin B2	578.1418	579.15	C_30_H_26_O_12_	18	3.84	10.79	427.09	Water *F. semicordata*	93.73
	578.1418	579.1504	C_30_H_26_O_12_		1.24	7.44	427.10, 409.09, 139.03, 127.03, 123.04, 163.03, 247.06	*T. chebula* (bark)	
Ellagic acid-*O*-pentoside	434.0479	435.0578	C_19_H_14_O_12_	14	4.67	7.27	303.01	Water *C. operculatus*	—
Quinic acid	192.0628	193.07	C_7_H_12_O_6_	2	3.95	3.02	175.06, 157.05, 113.02	EA *F. semicordata*	—
	192.0628	193.0705	C_7_H_12_O_6_	—	0.53	1.05	—	Water *F. lacor*	—
Hydroxyl-6-gingerol-*O*-β-d-glucuronide	486.20	487.22	C_23_H_34_O_11_	7	4.90	12.60	311.15, 295.11	Water *F. semicordata*	—
3-*O*-Galloylnorbergenin	466.0700	467.0872	C_20_H_18_O_13_		1.37	11.18	171.03, 53.01	EA *C. operculatus*	—
3,4,5-Tri-*O*-galloylquinic acid	648.0915	649.1082	C_28_H_24_O_18_		1.76	10.17	495, 343	EA *C. operculatus*	—
Tri-*O*-methoxyellagic acid	392.0374	393.0435	C_17_H_12_O_11_		4.29	3.21	—	EA *C. operculatus*	—
Butyl isobutyl phthalate	278.1512	279.1601	C_16_H_22_O_4_	6	3.66	30.01	—	Water *C. operculatus*	—
	278.1512	279.1603	C_16_H_22_O_4_	6	4.72	29.94	—	Water *T. chebula* (fruits)	—
	278.1512	279.1590	C_16_H_22_O_4_	6	0.30	22.65	—	Water, EA *F. lacor*	—
	278.1512	279.1592	C_16_H_22_O_4_	6	0.40	18.64	—	*T. chebula* (bark)	84.29
Eudesmin	386.1723	387.1817	C_22_H_26_O_6_	10	3.98	17.45	—	Water *F. lacor*	—
Lasiodiplodin	292.1669	293.1736	C_17_H_24_O_4_	6	3.00, 3.22	24.28, 24.01	—	Water, EA *F. lacor*	—
1-Monopalmitin	330.2764	331.2842	C_19_H_38_O_4_	1	3.54, 0.72	27.76, 27.78	125.09	Water, EA *F. lacor*	—
α-Tocospiro B	462.3703	463.3794	C_29_H_50_O_4_	5	2.75	36.62	—	Water *F. lacor*	—

a EA: ethyl acetate, RDBE: ring double bond equivalents.

### GNPS-based metabolomic profiling

**Fig. 2:**
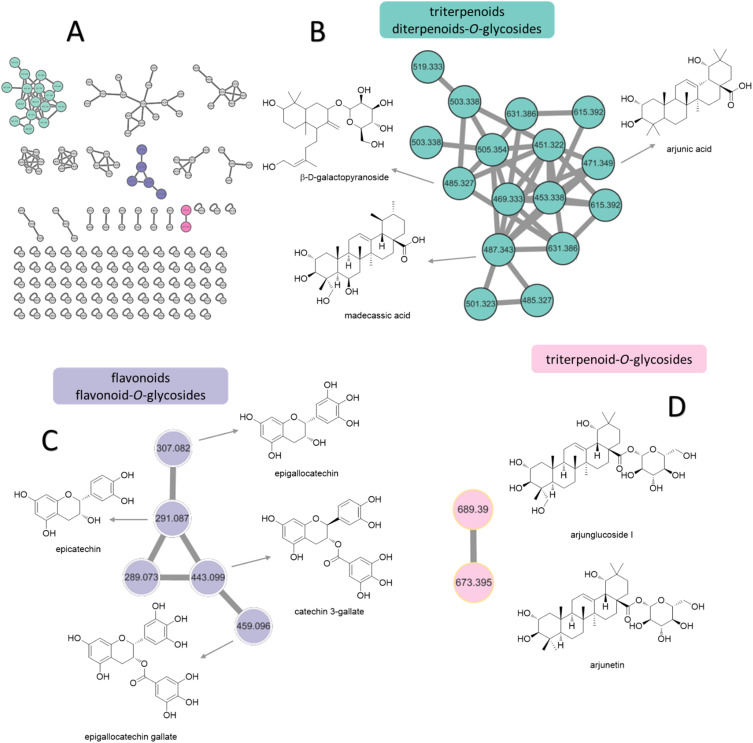
Molecular networking and identification of natural compounds from the extract of *T. chebula* bark. (A) Molecular networking and chart of categorized compounds in this study. (B) Zoomed-in molecular networking of triterpenoids and diterpenoids. (C) Zoomed-in molecular networking of flavonoids and flavonoid-*O*-glycosides. (D) Zoomed-in molecular networking of triterpenoid-*O*-glycosides.

Based on the anti-diabetic activity of the methanol extract and ethyl acetate fraction of the bark of *T. chebula*, a comprehensive and detailed phytochemical profile was investigated using MS/MS and a GNPS-based metabolomics platform. A total of 205 and 160 individual ion species (MS/MS spectra) were present as nodes in the network map for the methanol extract and the EA fraction, respectively. Moreover, 22 compounds were tentatively identified from *T. chebula* bark, belonging to various groups of phytochemicals including phenolic compounds, diterpenoids-*O*-glycosides, triterpenoids, triterpenoid-*O*-glycosides, flavonoids, flavonoid-*O*-glycosides, fatty acids, and ceramides (Fig. 2, Table 5).

**Table 5:** Details of the identified constituents in *T. chebula* bark fractions using MS/MS and GNPS analysis

Methanol (AT1) and EA (AT2) extract of the bark of *T. chebula*						Fractions
Comp. name	RT	*m*/*z*	MS^2^ fragmentation pattern	Molecular formula	Exact mass	
Epigallocatechin	4.87	307.07	181.04, 139.03	C_15_H_14_O_7_	306.07	AT2
Epicatechin	7.42	291.09	165.05, 139.04, 123.04	C_15_H_14_O_6_	290.08	AT1, 2
Catechin	7.44	291.09	162.05, 139.04, 123.04	C_15_H_14_O_6_	290.08	AT1, 2
Procyanidin B2	7.50	579.15	409.09, 287.05, 247.06, 163.04, 127.04	C_30_H_26_O_12_	578.14	AT1, 2
Arjunglucoside I	10.47	689.39	527.33, 185.04, 89.06	C_36_H_58_O_11_	666.40	AT1, 2
Arjunetin	11.66	673.39	511.34, 185.04	C_36_H_58_O_10_	650.40	AT1, 2
Sesamin	15.60	337.11	319.09, 289.08, 267.06, 203.08, 185.06, 135.04	C_20_H_18_O_6_	354.11	AT1
Epigallocatechin gallate	7.93	459.09	139.04	C_22_H_18_O_11_	458.08	AT2
Quercitrin	8.34	449.09	303.04, 85.02	C_21_H_20_O_11_	448.10	AT1, 2
Ellagic acid	8.53	303.01	291.09, 273.07, 153.02	C_14_H_6_O_8_	302.01	AT1
(−)-Catechin 3-gallate	8.93	443.09	273.08, 153.02, 123.04	C_22_H_18_O_10_	442.09	AT1, 2
Epicatechin gallate	8.99	443.10	273.07, 139.04, 123.04	C_22_H_18_O_10_	442.09	AT1, 2
Arjunic acid	11.66	471.35	407.33, 201.16, 107.08	C_30_H_48_O_5_	488.35	AT2
Madecassic acid	12.19	487.342	451.32, 187.15, 145.10, 119.08, 95.08	C_30_H_48_O_6_	504.35	AT1, 2
β-d-Galactopyranoside	12.25	485.31	199.15, 171.12, 145.10, 105.07, 81.07	C_26_H_44_O_8_	484.30	AT2
Phytosphingosine	15.38	318.30	282.28, 60.04	C_18_H_39_NO_3_	317.29	AT1
Palmitic acid	17.38	402.36	283.26	C_22_H_42_O_6_	402.30	AT1
Dibutyl phthalate	18.64	279.16	149.02, 121.03	C_16_H_22_O_4_	278.15	AT1, 2
Linolenic acid	20.53	279.23	109.10, 95.05, 81.07, 67.05	C_18_H_30_O_2_	278.22	AT1, 2
Oleanolic acid	20.74	439.36	249.18, 217.19, 191.18, 95.08	C_30_H_48_O_3_	456.36	AT1
Dioctyl phthalate	22.52	391.28	167.03, 149.02, 71.08, 57.07	C_24_H_38_O_4_	390.28	AT1
Erucamide	23.80	675.67	338.34, 321.31	C_22_H_43_NO	337.33	AT1, 2

Molecular networking analysis involves representing each metabolite as a node labeled with its *m*/*z* value. The metabolites were then grouped into several clusters based on the similarity of their fragmentation patterns, indicating similarities in their core chemical structures.^35^ From molecular networking and chart of categorized compounds (Fig. 2A), the major clusters included diterpenoid-*O*-glycosides, triterpenoids (Fig. 2B), flavonoid derivatives (Fig. 2C), and triterpenoid-*O*-glycosides (Fig. 2D).

The terpenoid clusters, characterized by precursor ions at *m*/*z* 471.3449, 485.3109, and 487.3403, were identified as arjunic acid, β-d-galactopyranoside, and madecassic acid, respectively. The flavonoids were annotated for *m*/*z* 291.0869 and 307.0736 clusters of epicatechin and epigallocatechin, respectively. Moreover, the neutral loss of 152 Da from both precursor ions at *m*/*z* 443.0947 and 459.0964 indicated the presence of gallate substitution, suggesting the presence of catechin gallate and epigallocatechin gallate. Finally, the networking of glycosylated triterpenoids, namely arjunglucoside I and arjunetin, was attributed to the presence of intense product ion signals at *m*/*z* 527.3334 and 511.3390, respectively, originating from the aglycone moiety. Additionally, the fragmentation patterns exhibited prominent neutral losses of 162 Da owing to the elimination of glucose. The various constituents observed in the two fractions from *T. chebula* bark are listed in Table 5 along with their *m*/*z* values in positive ion mode, retention times, MS/MS fragmentation patterns, and molecular formulas.

## Discussion

This study aimed to explore the secondary metabolites, TPC, TFC, and antioxidant activities of extracts of *C. operculatus*, *T. chebula*, *F. semicordata*, and *F. lacor*. In addition, the enzyme inhibitory activities of the different fractions against α-glucosidase and α-amylase were determined and are presented in Tables 2 and 3. The results suggest that methanolic extracts, ethyl acetate, and water fractions showed potent activity against diabetic enzymes, which might be due to the synergistic effects of different chemical constituents. The ethyl acetate fractions exhibit a high flavonoid content, thereby attributing to their role in the inhibition of enzymes.^36,37^ Previous studies have also suggested that the methanolic, ethyl acetate, and water fractions significantly inhibit the activities of both α-glucosidase and α-amylase, respectively.^32^

**Fig. 3:**
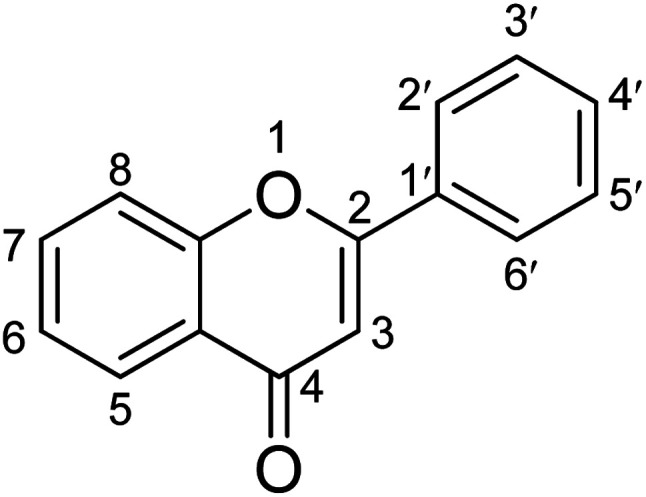
Structure–activity relationship of α-glucosidase inhibitory activity of flavonoids.

High blood glucose levels lead to the formation of free radicals that damage macromolecules within the cell and increase the risk of complications related to hyperglycemia. Thus, compounds with antioxidant properties can aid in the mitigation of diabetes-related problems.^38^ Phenolic compounds are an important class of secondary metabolites with antioxidants as well as antidiabetic activity. Polyphenols with several hydroxyl groups can act as a source of hydrogen and electron donor to radicals to stabilize them and hence reduce oxidative stress which plays an important role in the mitigation of diabetes.^39^ The introduction of hydroxyl and methoxyl groups to different positions of flavonoid structures is the most common form in natural products. The patterns of hydroxyl and methoxyl groups on aryl rings A and B of flavonoids were reported to be closely associated with their biological activity towards enzymes. The hydroxylation of flavonoids at positions C3, C6, C3′, and C4′ all exhibited better inhibitory activity on α-glucosidase than the unhydroxylated forms at the corresponding positions.^39^ The antidiabetic effect of flavonoids also can be increased by replacing the hydroxyl groups at the C3 position with various functional group moieties, such as methoxy, sugar-like rhamnose, galloyl group, and chlorine atom. However, the glycosyl and geranyl moieties at C7 can suppress the antidiabetic effect. The presence of a C2=C3 double bond, along with the C4-oxo group and aryl ring A, is essential for maintaining the planar structure of flavonoids. Hydrogenation of this double bond weakens the enzyme's inhibitory activity likely because the planar molecular structure significantly influences the binding conformation within the enzyme's active site (Fig. 3).^40,41^

Among four selected medicinal plants, *T. chebula* (bark) has shown excellent free radical scavenging activity with IC_50_ values of 5.144 ± 0.06 μg mL^−1^ corresponding to control quercetin (6.3 ± 1.00 μg mL^−1^). This may be due to the presence of high TPC and TFC values in *T. chebula.* Such promising antioxidant potential could be ascribed to ellagic acid (IC_50_ = 2.012 ± 0.10 μg mL^−1^), catechin (IC_50_ = 6.7 μM), epicatechin (IC_50_ = 6.8 μM),^42^ arjunglucoside I, quercitrin, flavogallonic acid,^43^ bartogenic acid and arjunic acid identified in the *T. chebula* bark extracts.

Since α-glucosidase and α-amylase enzymes are thought to be promising targets for the treatment of diabetes mellitus,^44^ we assessed the antidiabetic potential of four medicinal plants. The promising inhibitory activity towards α-glucosidase was observed in the water fraction of *T. chebula* (fruits) with an IC_50_ value of 1.07 ± 0.01 μg mL^−1^ followed by methanolic and water fraction of *C. operculatus* (IC_50_ = 1.26 ± 0.03 μg mL^−1^ and IC_50_ = 4.91 ± 0.02 μg mL^−1^ respectively). Moreover, the water fraction of *F. semicordata* (IC_50_ = 5.0 ± 0.2 μg mL^−1^) and ethyl acetate fraction of the *T. chebula* bark (IC_50_ = 6.633 ± 1.82 μg mL^−1^) displayed excellent activity as compared to acarbose (positive control, IC_50_ = 344.23 ± 1.03 μg mL^−1^). The high polyphenol and flavonoid contents in these plant extracts may be responsible for their promising α-glucosidase-inhibitory activity. Previously, α-glucosidase inhibitory activity was observed with an IC_50_ value of 38.2 μg mL^−1^ (methanolic extract), and 9.0 μg mL^−1^ (water extract) for *T. chebula*, 68.2 ± 3.4 μg mL^−1^ for *C. operculatus*,^14^ 4.226 ± 0.43 μg mL^−1^ (ethyl acetate) for *F. semicordata*.^45^

Similarly, the significant α-amylase inhibitory activity was shown by the methanolic extract of *F. semicordata* (IC_50_ = 46.8 ± 1.8 μg mL^−1^) followed by water and methanolic extract of *C. operculatus* (53.2 ± 1.0 μg mL^−1^ and 62.49 ± 1.89 μg mL^−1^ respectively) corresponding positive control acarbose (6.1 ± 0.10 μg mL^−1^). In previous work, α-amylase inhibitory activity was observed in ethyl acetate fraction of *F. semicordata* and *T. chebula* extract with IC_50_ values of 4.861 ± 0.41 and 15.1 ± 1.4 μg mL^−1^ respectively.^45,46^ The variation between our results and the literature data might be attributed to various factors, including the degree of ripeness at the time of harvesting, environmental factors, processing, and storage.^47^

The MS-based metabolomics approach was employed in our study to putatively identify all the secondary metabolites present in the plant extracts responsible for the anti-diabetic activity. Most of the compounds annotated in our study were similar to those in the literature; the ethyl acetate extract of *T. chebula* showed a base peak at 357.0455 [M + H]^+^ and fragment peaks at 339, 321, 293, and 203 corresponding to chebulic acid.^48^ It has been reported as a potent compound for preventing the vascular complications associated with diabetes.^49^ The base peak at 371.0619, with fragment peaks at 191, 163, and 145, was predicted to be 2-*O*-caffeoyl hydroxy citric acid, which correlates with the fragmentation pattern reported in the literature.^50^ The molecular formula C_14_H_6_O_8_, with a base peak of 303.0140, has been proposed to be ellagic acid based on a comparative analysis of the spectral findings of Wu *et al.*^51^ As per the existing literature, ellagic acid demonstrates anti-diabetic effects by targeting the β-cells in the pancreas.^52^ Similarly, [M + H]^+^ at *m*/*z* 619.0970 along with a fragment peak at 153 was annotated as trigalloevaloglucosan IX based on the results of Abu-Reidah *et al.*^53^ Likewise, molecular ion [M + H]^+^ at *m*/*z* 385.07 was annotated as 2-*O*-feruloyl hydroxy citric acid with fragment peaks at 385, 209, and 195. The spectral data were consistent with the literature.^50^

A molecular ion at *m*/*z* 667.4054 [M + H]^+^ detected at retention time 10.33 min was identified as arjunglucoside I in *T. chebula* bark, isolated previously from *T. arjuna*.^54^ Previous study indicates that arjunglucoside I exhibits potential antidiabetic effects against the α-glucosidase enzyme (IC_50_ = 1074 ± 32 μM).^55^ Similarly, a protonated ion detected at *m*/*z* 657.3627 [M + H]^+^ was tentatively identified as galloylterminolic acid which was reported already in *T. albida*.^56^ Another molecular ion at *m*/*z* [M + H]^+^ was identified as flavogallonic acid, and was consistent with the literature found in the negative ESI-mode of *T. chebula*.^57^ The results of HPLC-MS analysis of *T. chebula* (fruits) showed a protonated ion with *m*/*z* 323.0762 [M + H]^+^ and sodiated ion as adduct at *m*/*z* 345.0580 [M + Na]^+^. Based on the literature, this compound was putatively identified as leucodelphidin.^58^ Arjunolic acid, previously identified in the *T. arjuna*,^59^ was also identified in (+)-ESI-MS/MS analysis at *m*/*z* 489.3570 [M + H]^+^. Against α-glucosidase isolated from *S. cerevisiae*, arjunolic acid demonstrated anti-diabetic activity (IC_50_ = 18.63 ± 0.32 g mL^−1^).^60^ In addition, bartogenic acid was annotated for a molecular ion at *m*/*z* 519.3313 [M + H]^+^. This compound has demonstrated anti-diabetic properties against intestinal-glucosidase (IC_50_ = 168.09 μg mL^−1^).^61^ Another molecular ion at *m*/*z* 505.3529 [M + H]^+^ was identified as arjungenin, previously reported by Honda and coworkers.^54^ It has been reported to show moderate antibacterial activity and beta-glucuronidase inhibitor activity.^62,63^ From *T. chebula* bark extract, a molecular ion with *m*/*z* 443.0978 [M + H]^+^ was tentatively annotated as (−)-epicatechin-3-*O*-gallate which was already reported by Singh *et al.*^64^ Based on a literature survey, gambiriin B1 was identified as a molecular ion with *m*/*z* 563.1549 [M + H]^+^ in the *T. chebula* bark.^65^ Similarly, rotundioic acid was isolated and identified.^66,67^ These data are consistent with our results, molecular ion peaks at 503.3363 [M + H]^+^ for rotundioic acid and 273.0759 [M + H]^+^ for butin. From the data obtained from the extract of *C. operculatus* and *T. chebula* bark, a protonated ion was detected at *m*/*z* 307.0815 and putatively identified as epigallocatechin or gallocatechin, which is similar to the study carried out by Lee and colleagues.^68^ Its efficacy for the treatment of prostate cancer is being currently studied and completed in phase 2 trial.^69^ It has been reported that epigallocatechin modifies glucose and lipid metabolism in rat hepatoma cell line H4IIE and notably enhances glucose tolerance in diabetic rodents.^70^

The compound exhibiting a base peak of 465.1023 and fragment peaks at 303 and 137 was annotated as isoquercitrin sourced from *F. lacor*.^71^ Isoquercitrin is currently being studied in a phase 2 clinical trial for its efficacy on Coronavirus Disease 2019.^72^ Moreover, it has been reported to demonstrate a prophylactic impact on diabetes mellitus.^73^ Similarly, from *F. semicordata* and *T. chebula* (bark) molecular ions at *m*/*z* 579.1504 and at *m*/*z* 291.0866 were annotated as procyanidin B2 and catechin based on the result analysis.^74,75^*In vitro*-glucosidase activity showed that procyanidin B2 could be used to reduce blood glucose levels.^76^ Whereas, in the α-glucosidase inhibitory assay, it has been found that catechin has possessed significant antidiabetic activity (IC_50_ = 87.55 ± 2.23 μg mL^−1^).^77^ We annotated quinic acid from *F. semicordata* and *F. lacor* with a protonated ion at *m*/*z* 193.0705 which was similar to those reported in the literature.^78^ A previous study showed that quinic acid potentially stimulates insulin secretion and enhances glucose tolerance.^79^ Hydroxyl-6-gingerol-*O*-β-d-glucuronide was annotated for a protonated ion with *m*/*z* 487.22 from the extract of *F. semicordata* with comparison to the spectral results from Zeng *et al.*^80^ The compound with molecular ion at *m*/*z* 435.0578 [M + H]^+^ was tentatively identified as ellagic acid-*O*-pentoside from *C. operculatus* based on the results obtained by Di Stefano and co-workers.^81^ Similarly, a compound with protonated ion at *m*/*z* 467.0872 [M + H]^+^ was identified as 3-*O*-galloylnorbergenin, which was previously reported.^53^ Moreover, molecular ions at *m*/*z* 649.1082 and 393.0435 were annotated as 3,4,5-tri-*O*-galloylquinic acid and tri-*O*-methoxyellagic acid, respectively from *C. operculatus*, as reported by Bindra *et al.* and Maldini *et al.*^82,83^ A molecular ion at *m*/*z* 279.1601 [M + H]^+^ and sodiated ion as adduct at *m*/*z* 301.1 [M + Na]^+^ was tentatively annotated as butyl isobutyl phthalate from *C. operculatus*, *T. chebula*, and *F. lacor.* This was previously reported by Bu *et al.* through LC/MS analysis, which was further supported by ESI-MS and NMR analysis.^84^ It was observed that butyl isobutyl phthalate exhibits a hypoglycemic effect when tested in living organisms, and therefore, acts as a non-competitive inhibitor of α-glucosidase (IC_50_ = 10.6 ± 1.1 μg mL^−1^).^84^ Likewise, molecular ion at *m*/*z* 387.1817 with retention time at 17.45 min was tentatively annotated as eudesmin from the water fraction of *F. lacor* and was already reported.^85^ Furthermore, based on literature data, a molecular ion at *m*/*z* of 293.1736 [M + H]^+^ was annotated as lasiodiplodin,^86^ while the compound analyzed in the ESI-MS (+) mode molecular ion at *m*/*z* 331.2842 was tentatively identified as 1-monopalmitin.^87^ Similarly, molecular ion at *m*/*z* 463.3794 was putatively identified as α-tocospiro B from the water fraction of *F. lacor*, as per the analysis performed by Chiang *et al.*^88^

GNPS-based molecular networking can be performed to validate manually annotated compounds and aid in the exploration of secondary metabolite derivatives according to similarities in the core chemical structure.^89^ Hence, this approach improves the visualization of the chemical constituents present in biological samples, forming clusters in molecular networking. In this study, the methanolic extract and EA fraction of the bark of *T. chebula* was subjected to comprehensive phytochemical profiling using MS/MS and a GNPS-based metabolomics platform, resulting in the identification of 22 compounds based on GNPS analysis and previous reports. Among these, 11 compounds, including linolenic acid, phytosphingosine, sesamin, dioctyl phthalate, palmitic acid, oleanolic acid, quercitrin, epigallocatechin gallate, arjunic acid, β-d-galactopyranoside, madecassic acid, and arjunetin, were annotated based on GNPS that were not identified by manual annotation. These compounds were categorized into three distinct molecular network clusters: diterpenoid-*O*-glycosides, triterpenoids, flavonoid derivatives, and triterpenoid-*O*-glycosides clusters (Fig. 2B–D). All compounds were previously reported to be isolated from *T. chebula*, indicating that further investigation is necessary for the unknown nodes and edges of *T. chebula*.

## Conclusion

Since ancient times, many traditional plants have been used by various ethnic groups to manage diabetes. Consequently, their scientific exploration is necessary. Four ethnically selected plants *C. operculatus*, *T. chebula*, *F. lacor*, and *F. semicordata,* exhibited significant enzyme inhibitory activity. In addition, LC/MS-based metabolomics and GNPS-based molecular networking were employed to explore and correlate secondary metabolites with the observed activities in these medicinal plants. Metabolic profiling revealed the presence of different classes of bioactive metabolites, including phenolic compounds, diterpenoids-*O*-glycosides, triterpenoids, triterpenoid-*O*-glycosides, flavonoids, flavonoid-*O*-glycosides, fatty acids, and ceramides. Additional investigation is recommended to isolate and identify potential inhibitors from the active fractions of plant extracts to develop a therapeutic candidate for diabetes and to explore the underlying mechanisms responsible for the observed anti-diabetic action.

## Author contributions

Niranjan Parajuli and Ki Hyun Kim designed and supervised the research; Arjun Prasad Timilsina, Bimal Kumar Raut, Chen Huo, and Rabin Budhathoki conducted computational metabolomics work; Bimal Kumar Raut, Arjun Prasad Timilsina, Karan Khadayat, and Niranjan Parajuli wrote the manuscript; Arjun Prasad Timilsina Prakriti Budhathoki, Mandira Ghimire and Karan Khadayat conducted *in vitro* assays; Niraj Aryal, Ki Hyun Kim, and Niranjan Parajuli edited the manuscript.

## Conflicts of interest

There are no conflicts of interest among authors.

## Supplementary Material

RA-013-D3RA04037B-s001
