# Supplementary material for: Metabolomics and molecular networking approach for exploring the anti-diabetic activity of medicinal plants
Source: RSC Adv. 2023 Oct 19;13(44):30665–79. doi: 10.1039/d3ra04037b (PMC10585453; doi:10.1039/d3ra04037b)
Supplement: RA-013-D3RA04037B-s001 [file RA-013-D3RA04037B-s001.pdf]

Electronic Supplementary Material (ESI) for RSC Advances.
This journal is © The Royal Society of Chemistry 2023

ARTICLE

Metabolomics and Molecular Networking Approach for Exploring the Anti-Diabetic Activity of Medicinal Plants

Received 00th January 20xx,
Accepted 00th January 20xx

DOI: 10.1039/x0xx00000x

Arjun Prasad Timilsina[†,a], Bimal Kumar Raut [†,a], Chen Huo [†,b], Karan Khadayat [a], Prakriti Budhathoki [a], Mandira Ghimire [a], Rabin Budhathoki [a], Niraj Aryal [c], Ki Hyun Kim *[b] , and Niranjan Parajuli *[a]

Content

1. Fig. S1: Mass spectrum of chebulic acid from *Terminalia chebula*;
2. Fig. S2: Mass spectrum of 2-*O*-caffeoyl hydroxycitric acid from *Terminalia chebula*;
3. Fig. S3: Mass spectrum of ellagic acid from *Terminalia chebula*;
4. Fig. S4: Mass spectrum of trigalloyllevoglucosan IX from *Terminalia chebula*;
5. Fig. S5: Mass spectrum of 2-*O*-feruloylhydroxycitric acid from *Terminalia chebula*;
6. Fig. S6: Mass spectrum of arjunglucoside I from *Terminalia chebula*;
7. Fig. S7: Mass spectrum of 23-galloylterminolic acid from *Terminalia chebula*;
8. Fig. S8: Mass spectrum of flavogallonic acid from *Terminalia chebula*;
9. Fig. S9: Mass spectrum of leucodelphidin from *Terminalia chebula*;
10. Fig. S10: Mass spectrum of arjunolic acid from *Terminalia chebula*;
11. Fig. S11: Mass spectrum of bartogenic acid from *Terminalia chebula*;
12. Fig. S12: Mass spectrum of arjungenin from *Terminalia chebula*;
13. Fig. S13: Mass spectrum of (-)-epicatechin-3-*O*-gallate from *Terminalia chebula*;
14. Fig. S14: Mass spectrum of gambiriin B1 from *Terminalia chebula*;
15. Fig. S15; Mass spectrum of rotundioic acid from *Terminalia chebula*;
16. Fig. S16; Mass spectrum of butin from *Terminalia chebula*;
17. Fig. S17: Mass spectrum of gallocatechin or epigallocatechin from *Cleistocalyx operculatus*;
18. Fig. S18: Mass spectrum of 4,4′-di-*O*-methylellagic acid from *Cleistocalyx operculatus*;
19. Fig. S19: Mass spectrum of isoquercitrin from *Ficus lacor*;
20. Fig. S20: Mass spectrum of catechin from *Ficus semicordata*;
21. Fig. S21: Mass spectrum of procyanidin B2 from *Ficus semicordata*;
22. Fig. S22: Mass spectrum of ellagic acid-*O*-pentoside from *Cleistocalyx operculatus*;
23. Fig. S23: Mass spectrum of quinic acid from *Ficus lacor*;
24. Fig. S24: Mass spectrum of hydoxyl-6-gingerol-*O*-β-D-glucuronide from *Ficus semicordata*;
25. Fig. S25: Mass spectrum of 3-*O*-galloylnorbergenin from *Cleistocalyx operculatus*;
26. Fig. S26: Mass spectrum of 3,4,5-tri-*O*-galloylquinic acid from *Cleistocalyx operculatus*;
27. Fig. S27: Mass spectrum of tri-*O*-methoxyellagic acid from *Cleistocalyx operculatus*;
28. Fig. S28: Mass spectrum of butyl isobutyl phthalate from *Cleistocalyx operculatus*;
29. Fig. S29 Mass spectrum of eudesmin from *Ficus lacor*;
30. Fig. S30: Mass spectrum of lasiodiplodin from *Ficus lacor*;
31. Fig. S31: Mass spectrum of 1-monopalmitin from *Ficus lacor*;
32. Fig. S32: Mass spectrum of α-tocospiro B from *Ficus lacor*;

[a.] *Biological Chemistry Lab, Central Department of Chemistry, Tribhuvan University, Kirtipur, Kathmandu, 44618, Nepal*
[b.] *School of Pharmacy, Sungkyunkwan University, Suwon 16419, Republic of Korea.*
[c.] *Department of Biology, University of Florida, Gainesville, FL 32611, USA*
[*.] *Corresponding author: niranjan.parajuli@cdc.tu.edu.np (N.P.), Tel: +977-1-4332034 (N.P.); khkim83@skku.edu (K.H.K.); +82-31-290-7700 (K.H.K.)*
† *These authors contributed equally to this work.*
Electronic Supplementary Information (ESI) available: [details of any supplementary information available should be included here]. See DOI: 10.1039/x0xx00000x

Please do not adjust margins

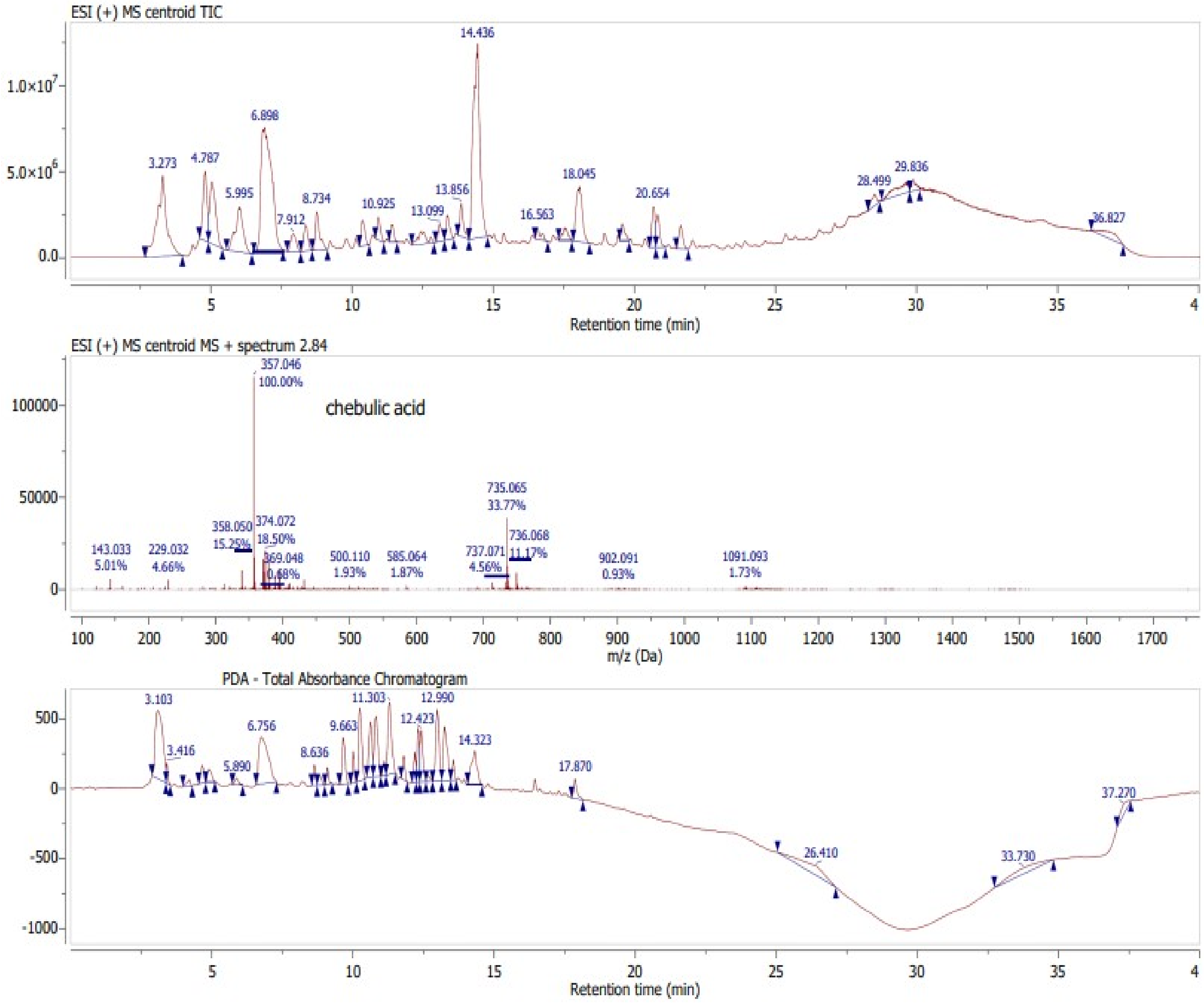

Fig. S1 Mass spectrum of chebulic acid from *Terminalia chebula*

This journal is © The Royal Society of Chemistry 20xx

Please do not adjust margins

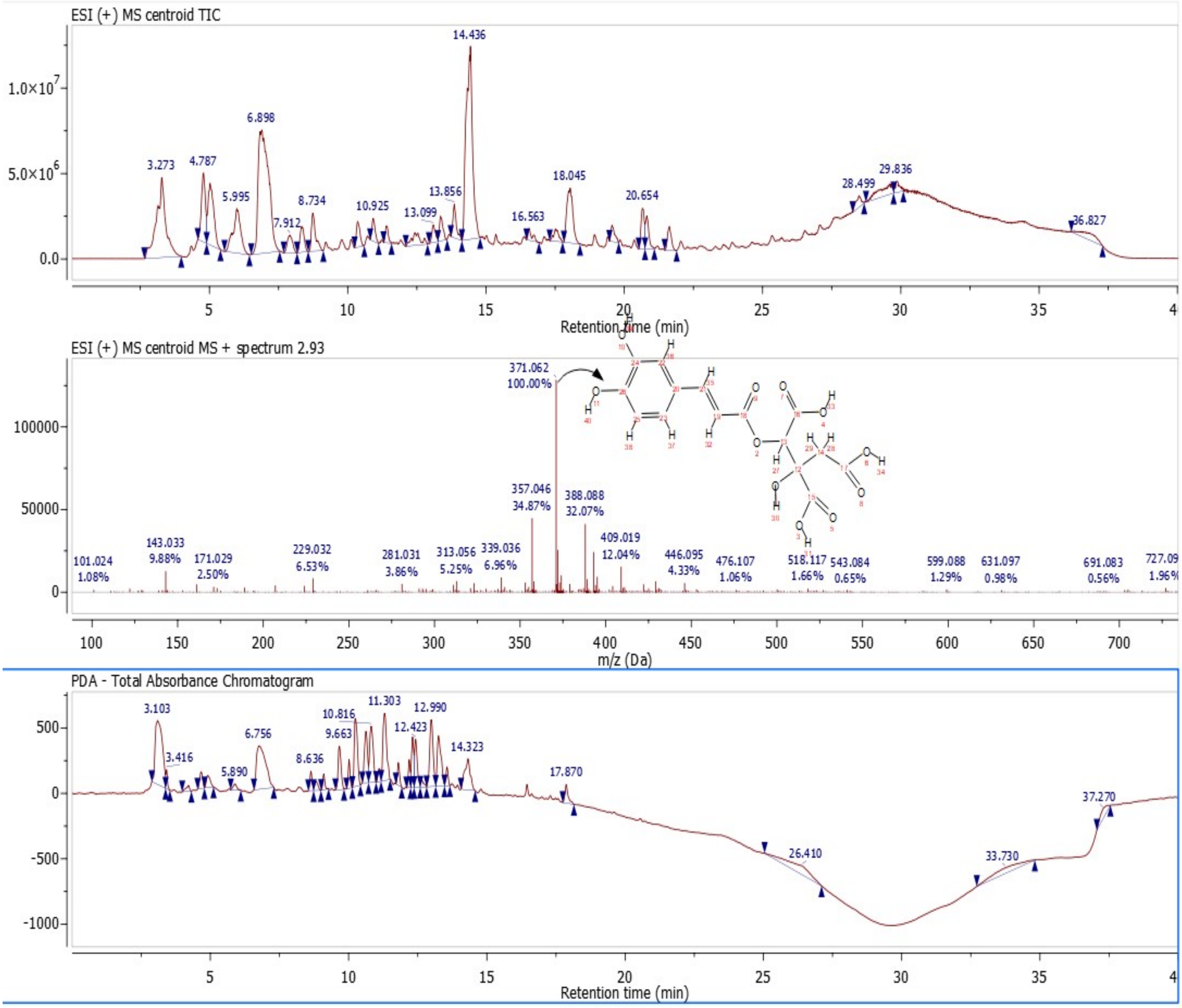

Fig. S2 Mass spectrum of 2-*O*-caffeoyl hydroxycitric acid from *Terminalia chebula*

Please do not adjust margins

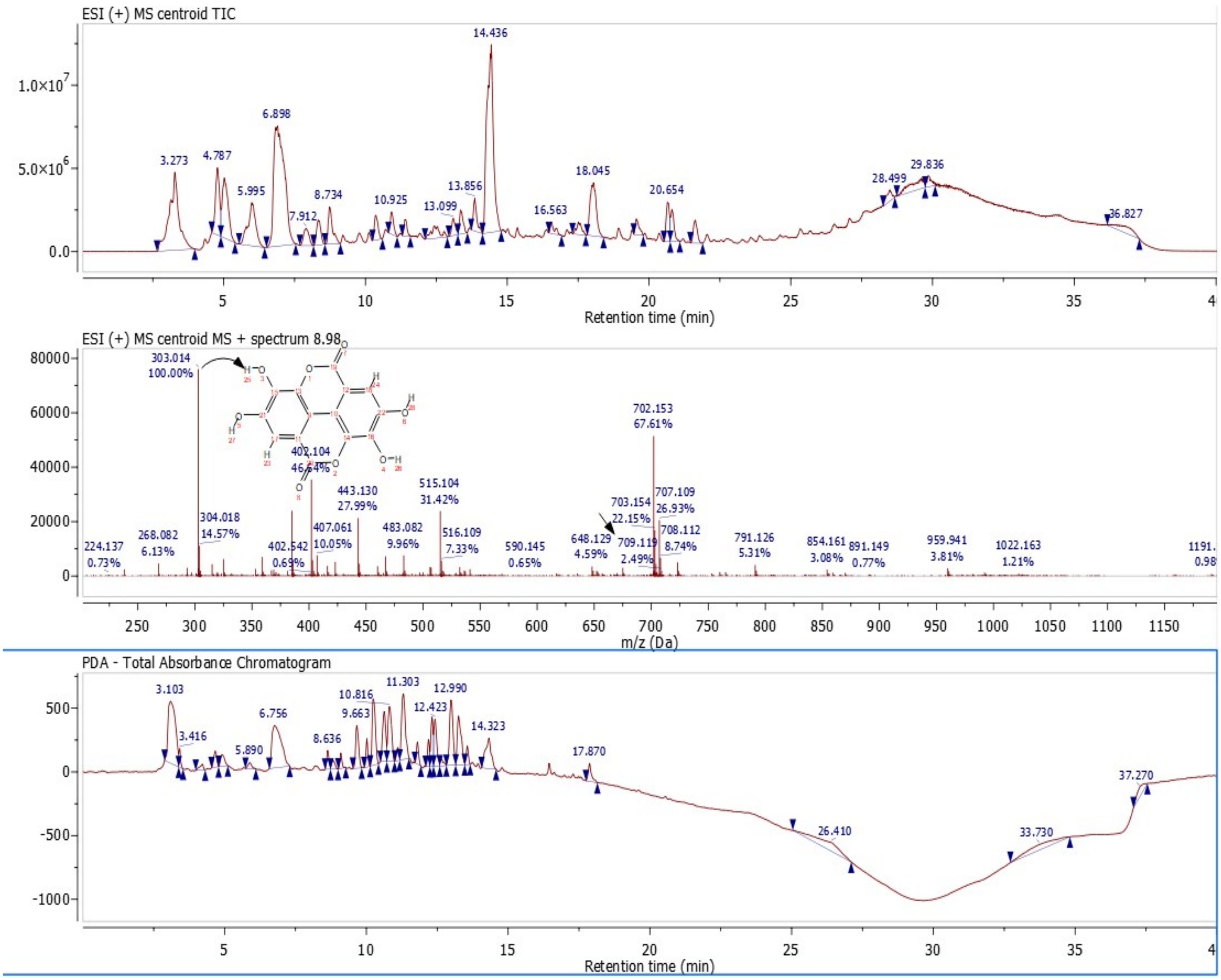

Fig. S3 Mass spectrum of ellagic acid from *Terminalia chebula*

This journal is © The Royal Society of Chemistry 20xx

Please do not adjust margins

Please do not adjust margins

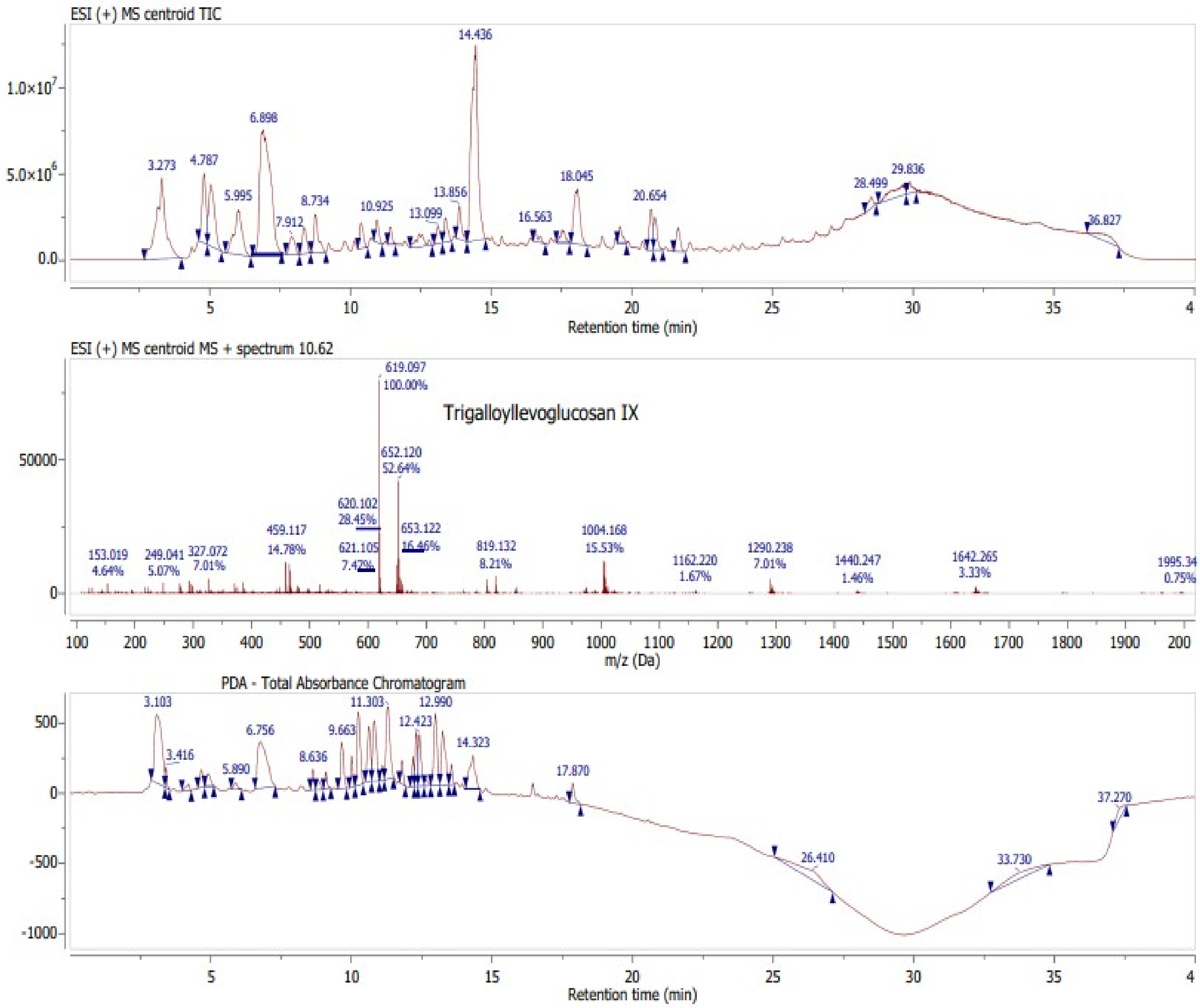

Fig. S4 Mass spectrum of trigalloyllevoglucosan IX from *Terminalia chebula*

Please do not adjust margins

Please do not adjust margins

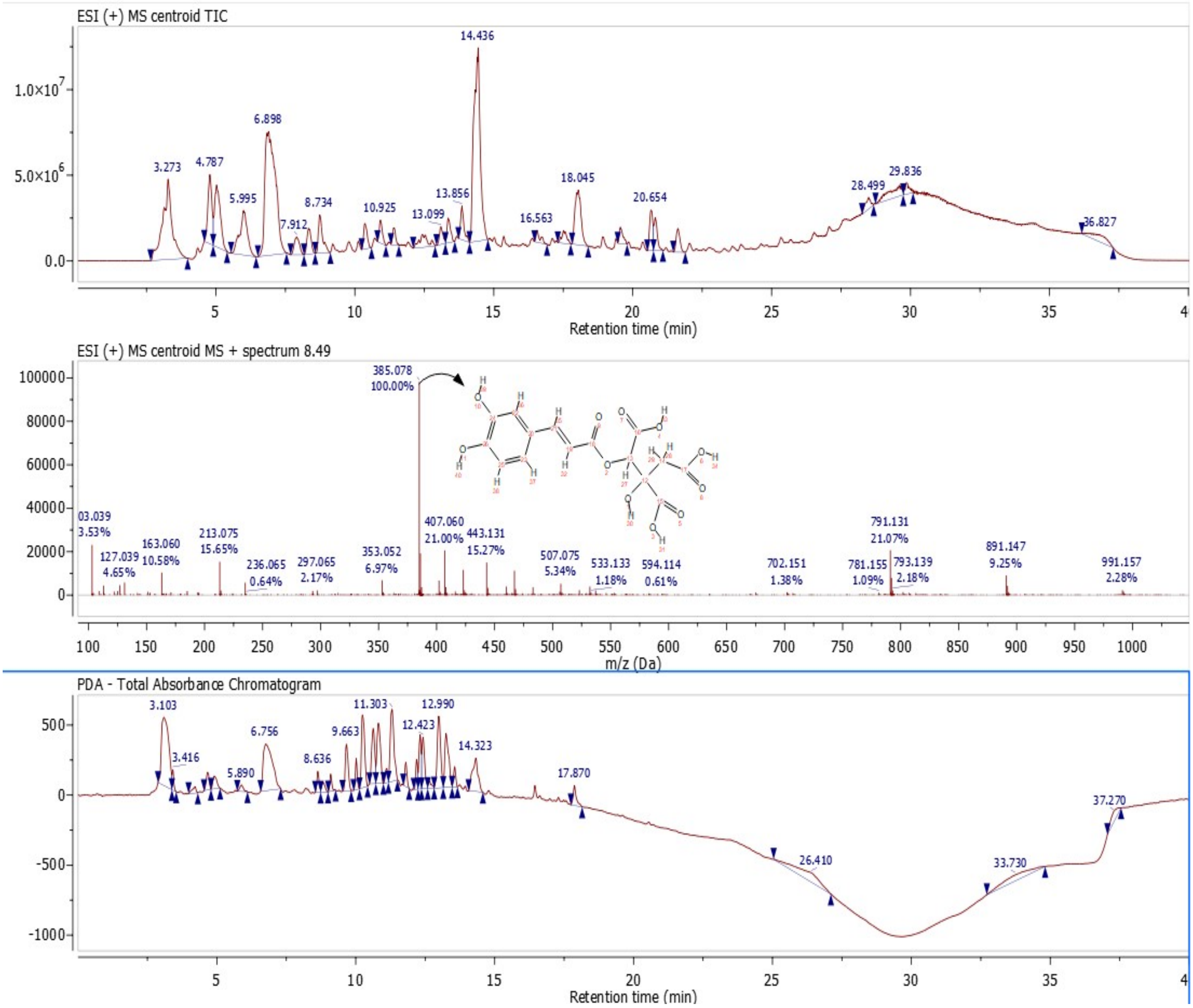

Fig. S5 Mass spectrum of 2-*O*-feruloylhydroxycitric acid from *Terminalia chebula*

This journal is © The Royal Society of Chemistry 20xx

Please do not adjust margins

Please do not adjust margins

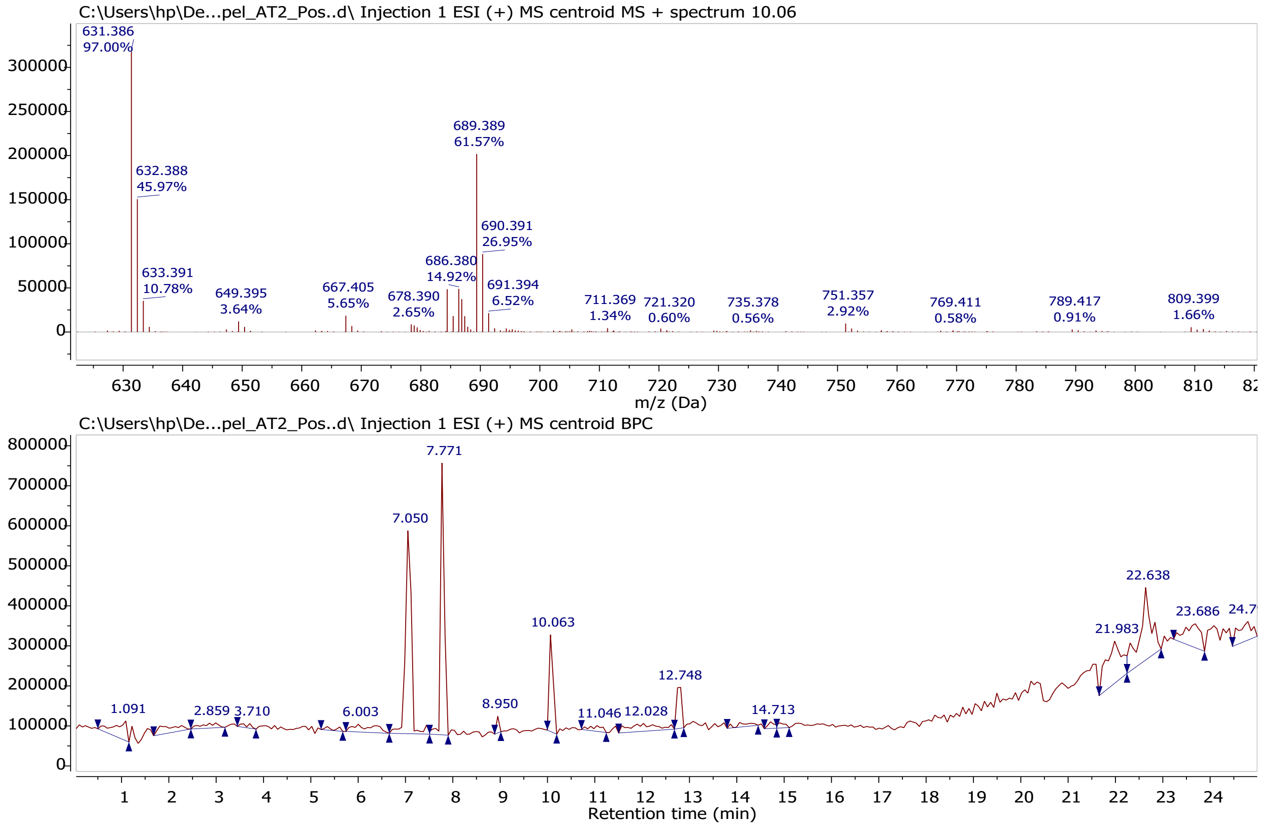

Fig. S6 Mass spectrum of arjunglucoside I from *Terminalia chebula* (bark)

Please do not adjust margins

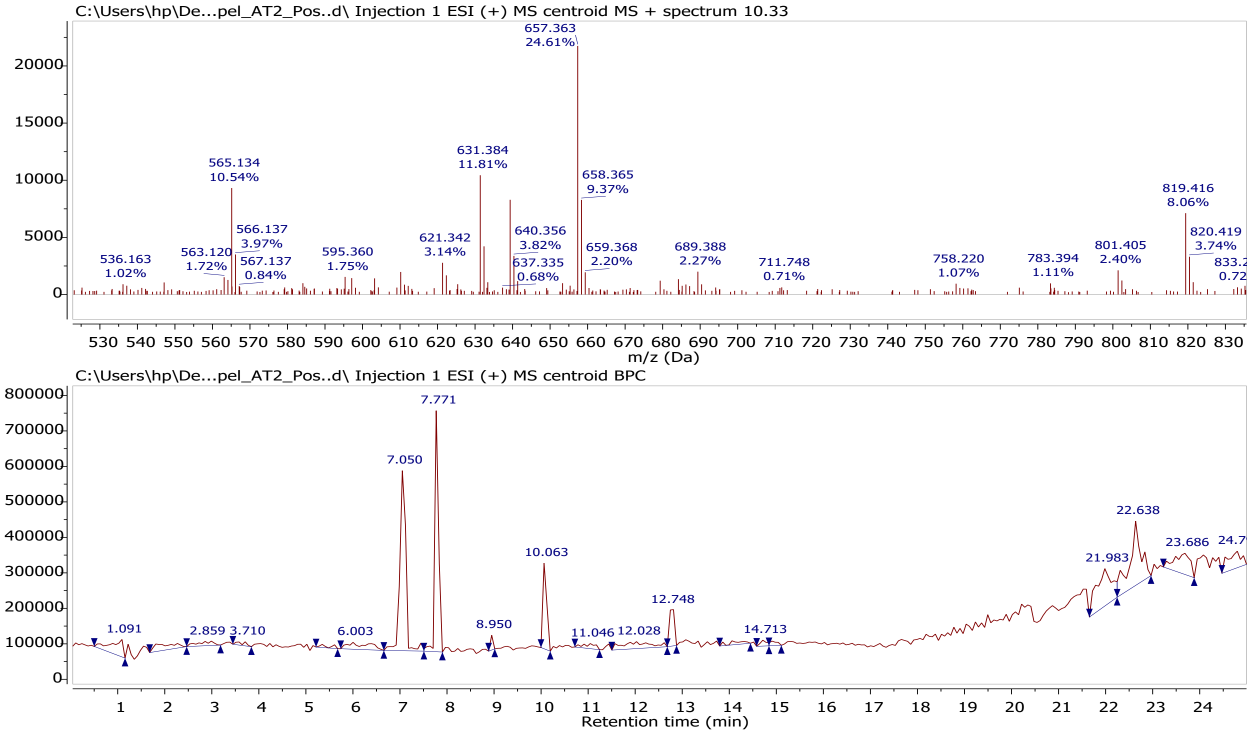

Fig. S7 Mass spectrum of 23-galloylterminolic acid from *Terminalia chebula* (bark)

Please do not adjust margins

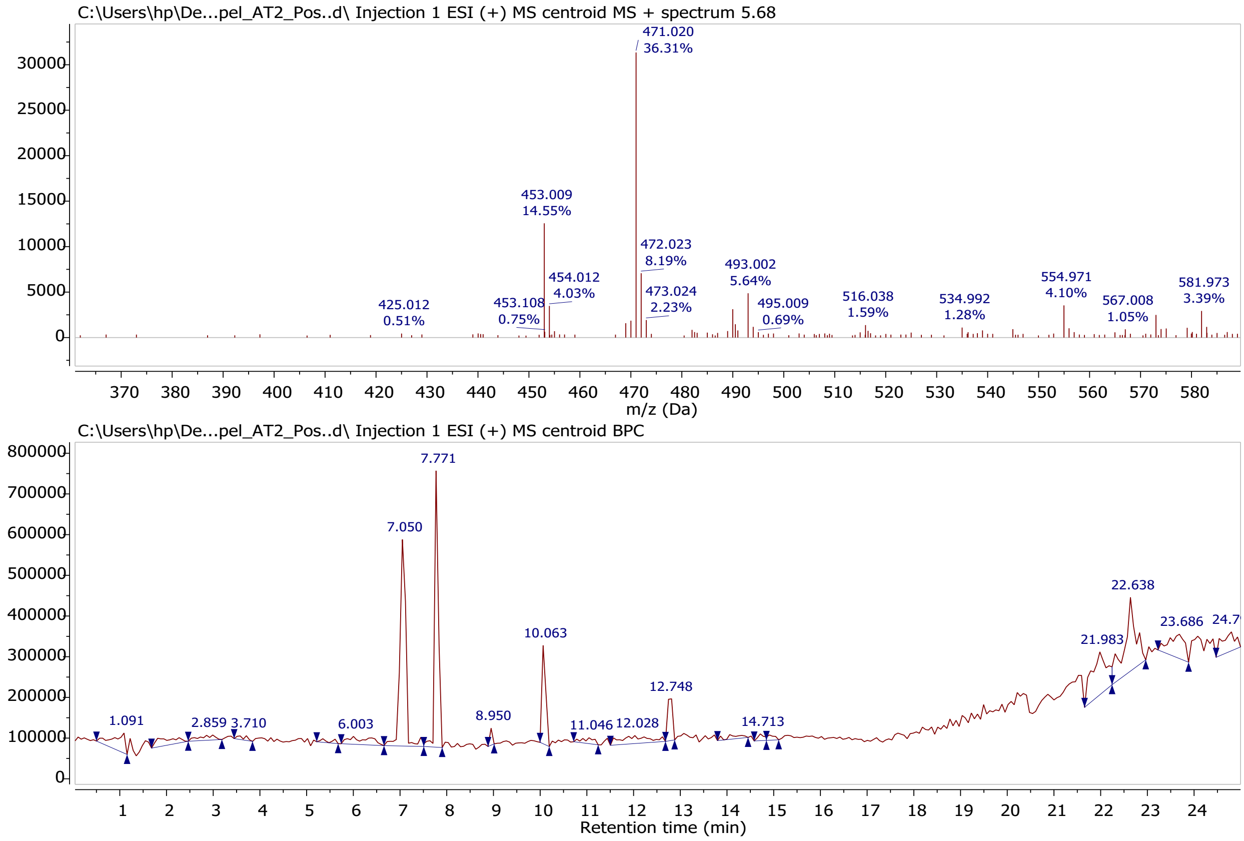

Fig. S8 Mass spectrum of flavogallonic acid from *Terminalia chebula* (bark)

Please do not adjust margins

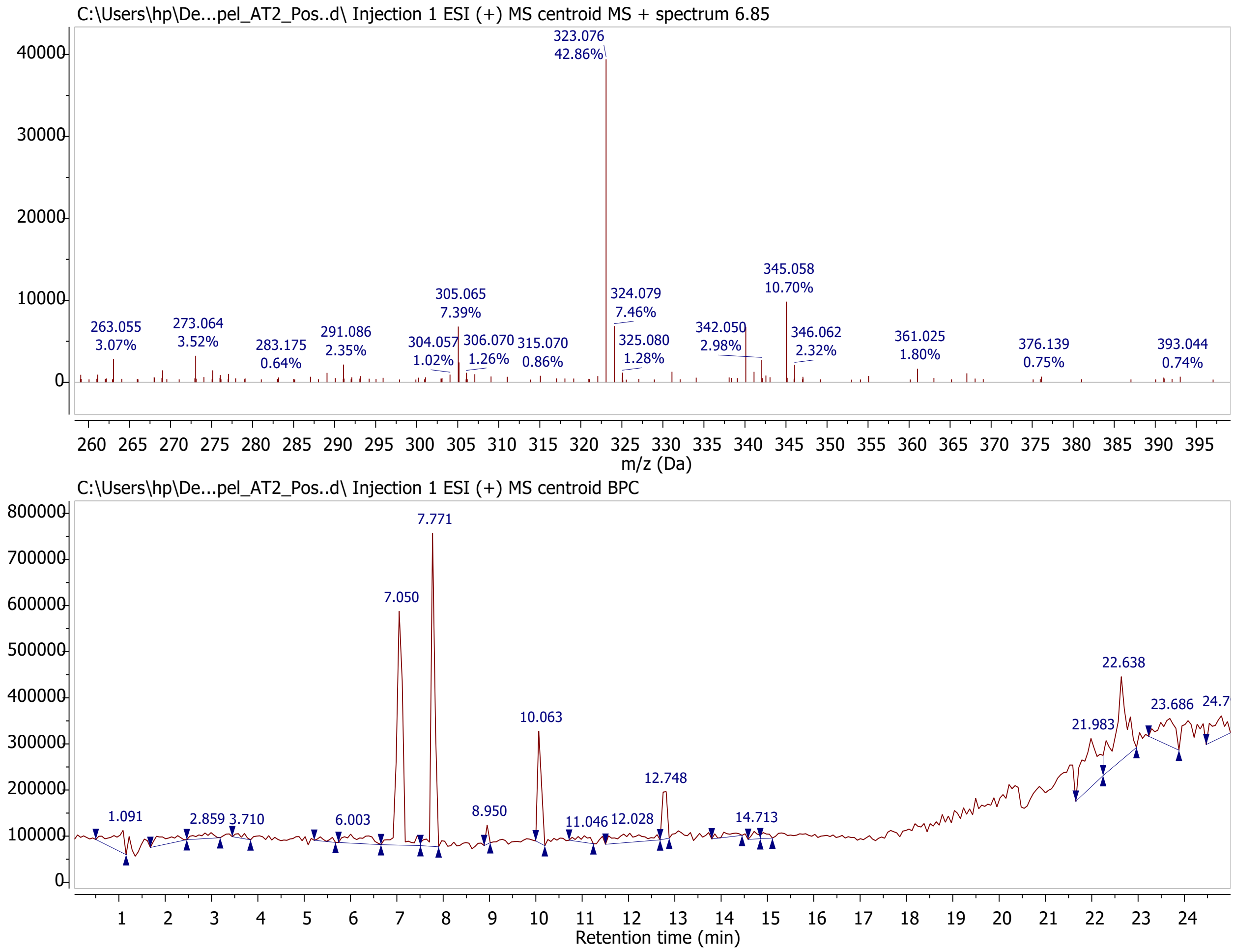

Fig. S9 Mass spectrum of leucodelphidin from *Terminalia chebula* (bark)

 This journal is © The Royal Society of Chemistry 20xx

Please do not adjust margins

Please do not adjust margins

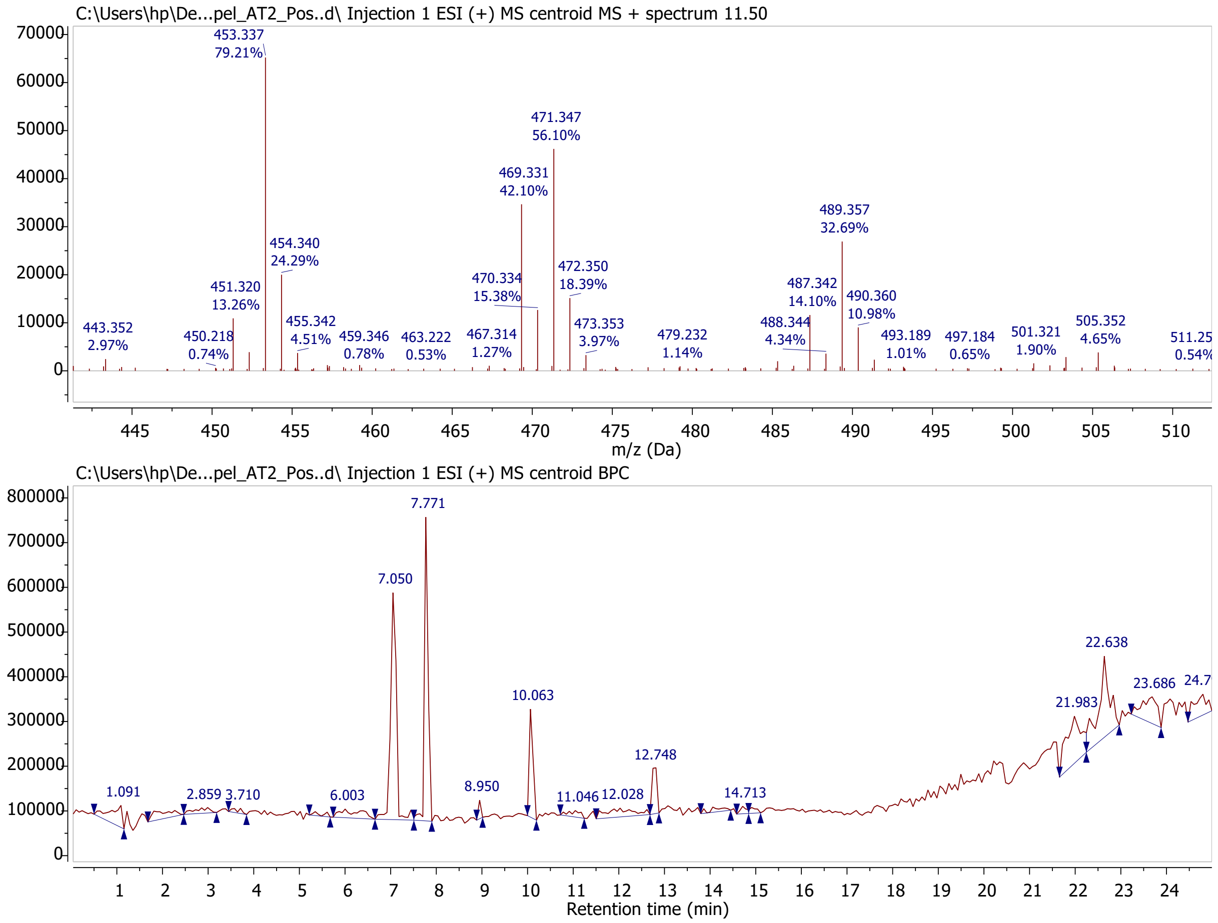

Fig. S10 Mass spectrum of arjunolic acid from *Terminalia chebula* (bark)

Please do not adjust margins

Please do not adjust margins

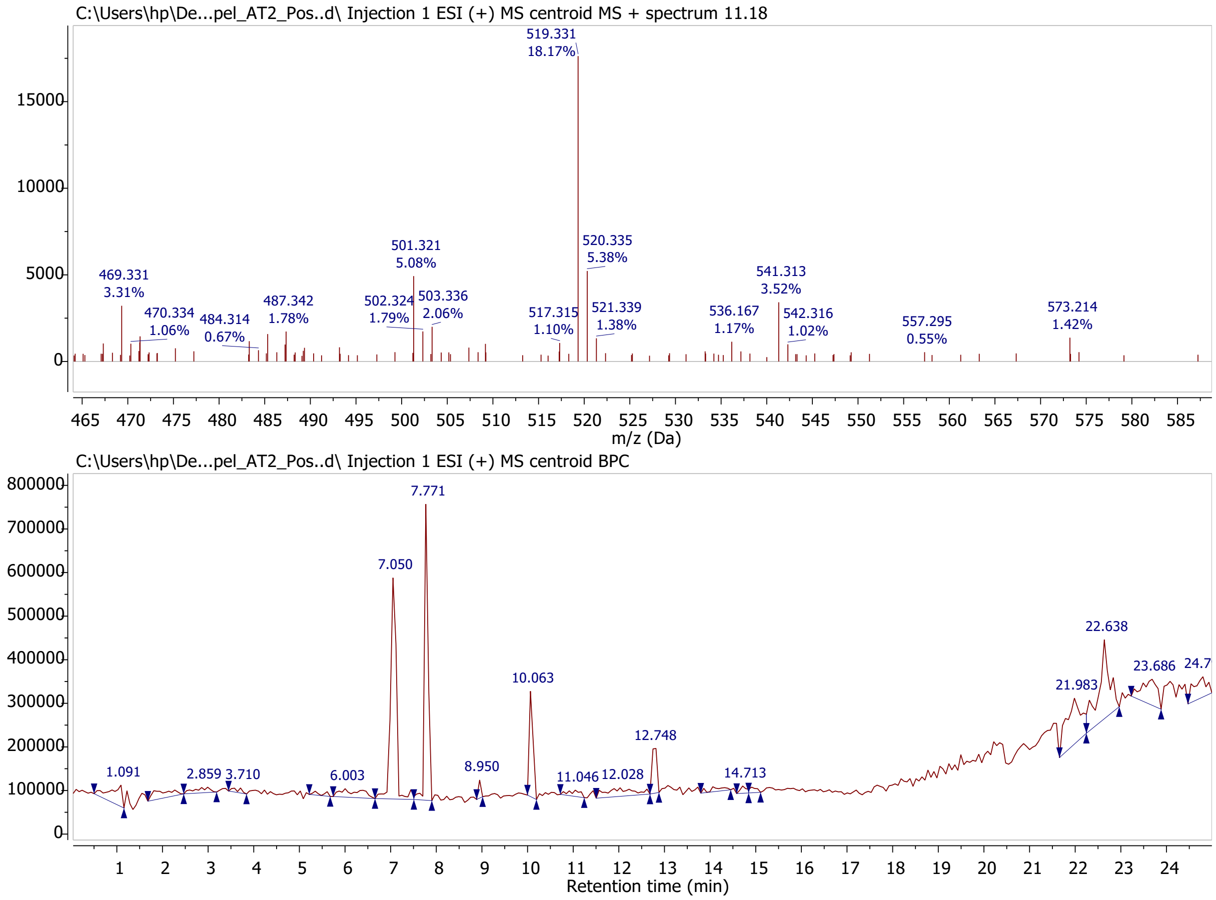

Fig. S11 Mass spectrum of bartogenic acid from *Terminalia chebula* (bark)

This journal is © The Royal Society of Chemistry 20xx

Please do not adjust margins

Please do not adjust margins

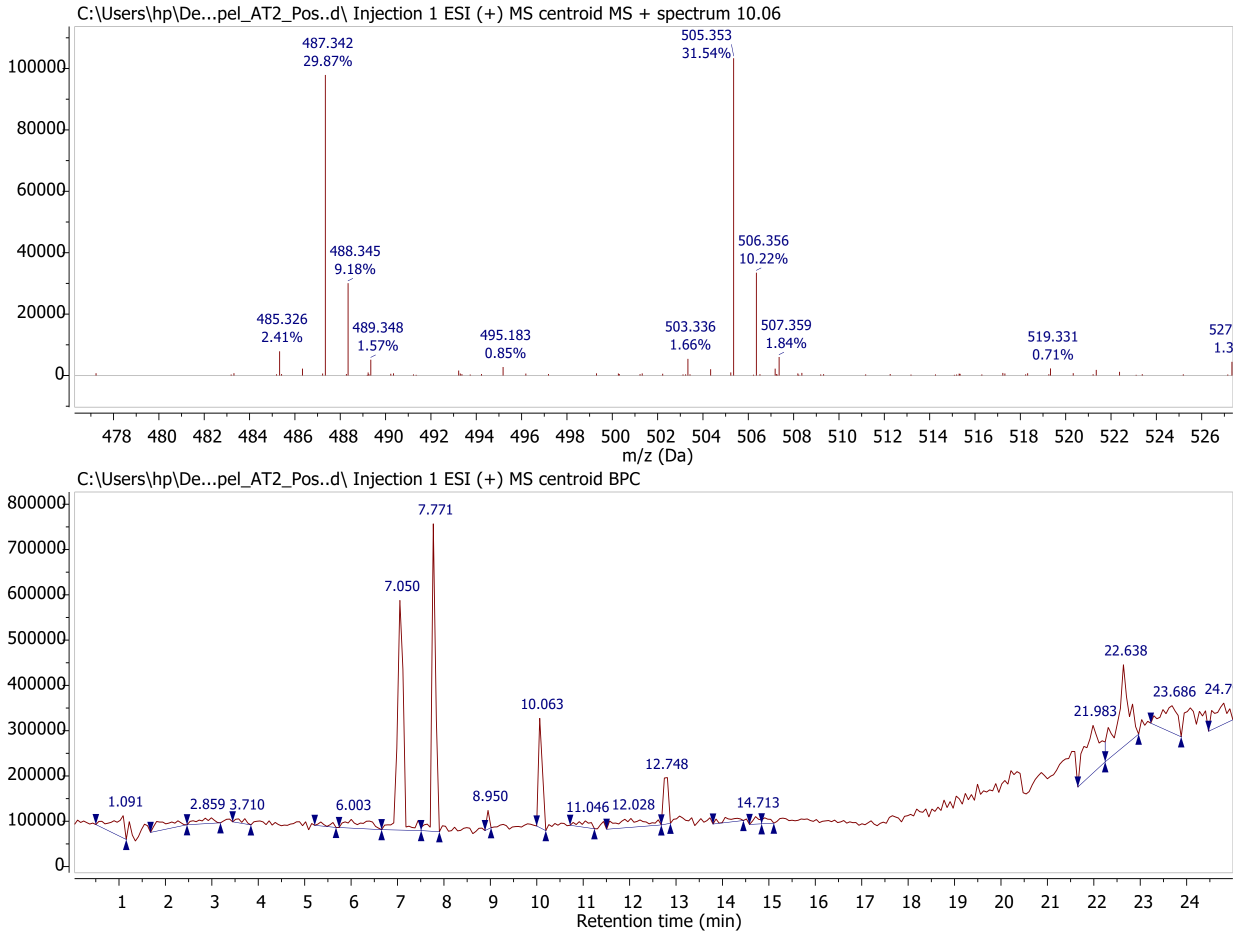

Fig. S12 Mass spectrum of arjungenin from *Terminalia chebula* (bark)

Please do not adjust margins

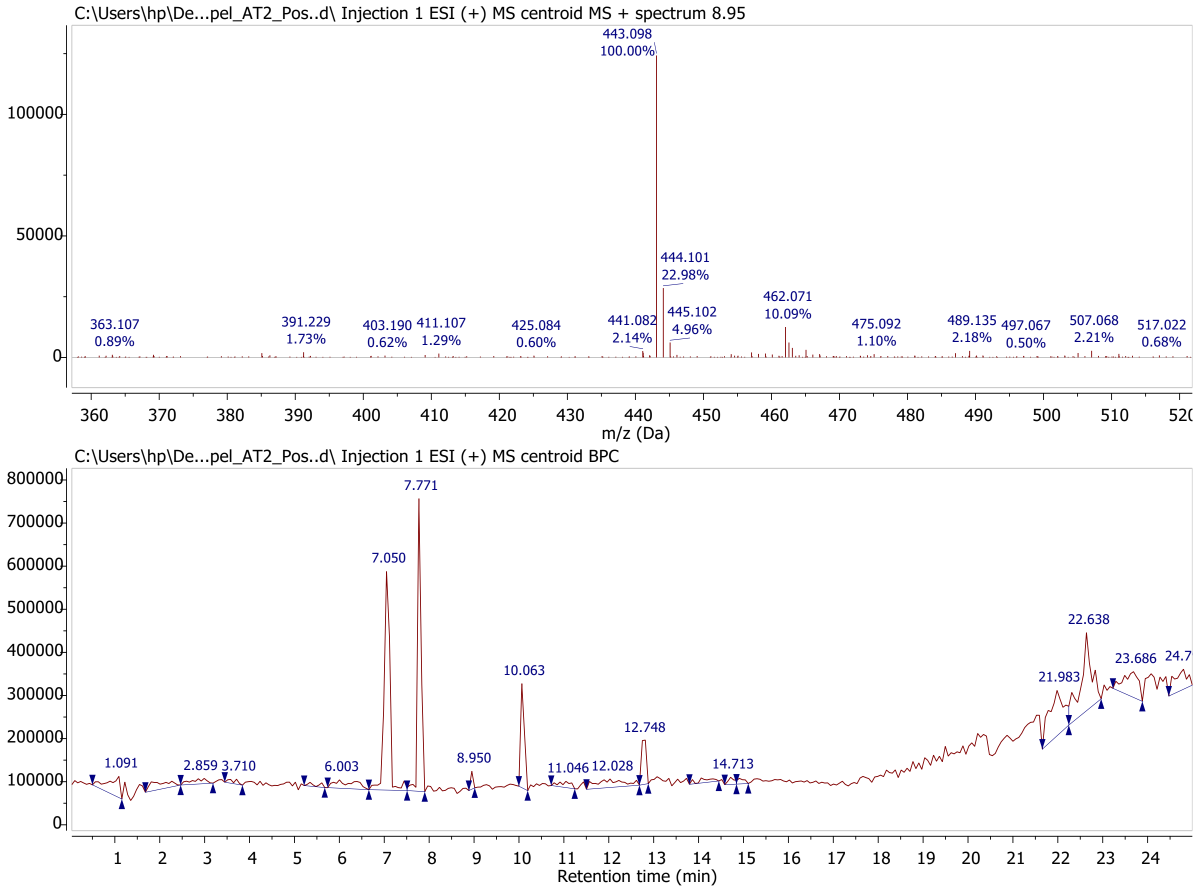

Fig. S13 Mass spectrum of (-)-epicatechin-3-*O*-gallate from *Terminalia chebula* (bark)

This journal is © The Royal Society of Chemistry 20xx

Please do not adjust margins

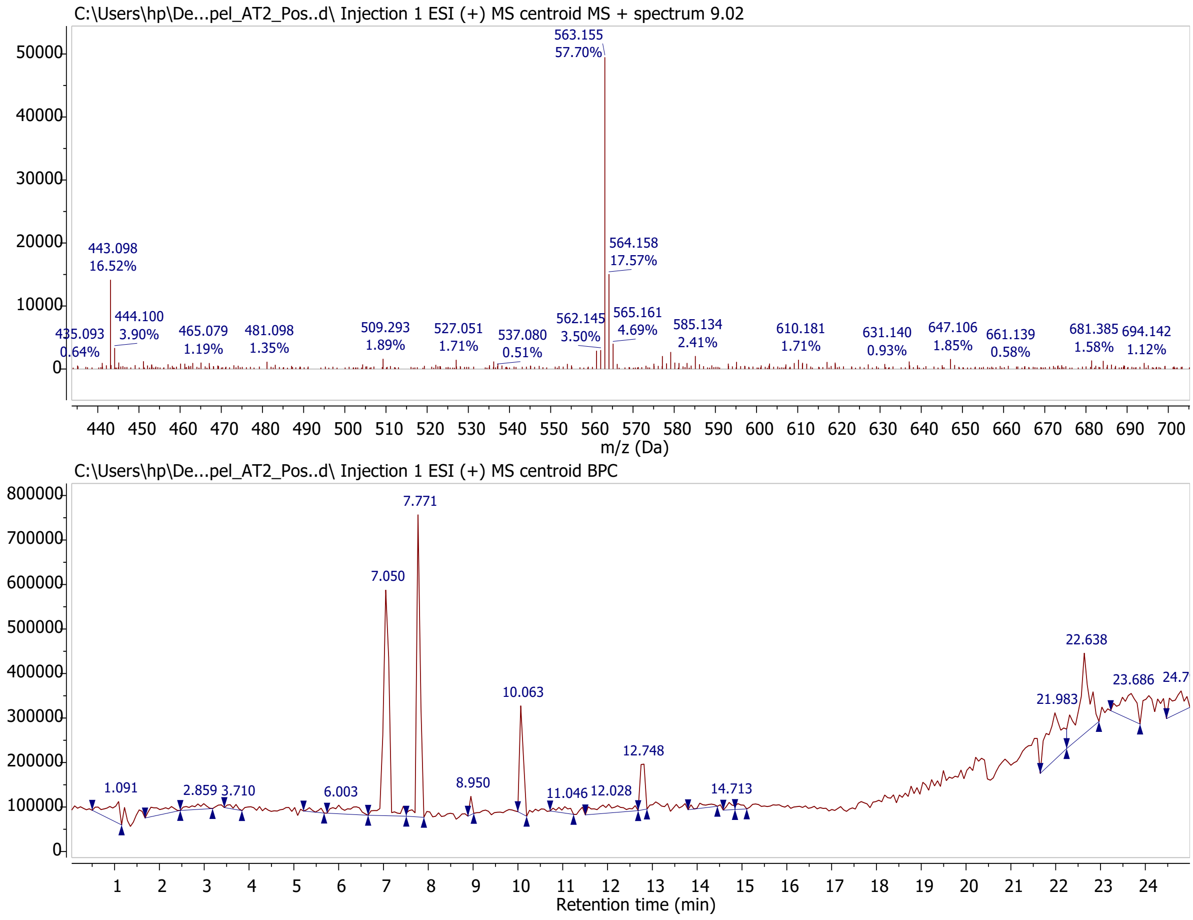

Fig. S14 Mass spectrum of gambiriin B1 from *Terminalia chebula* (bark)

Please do not adjust margins

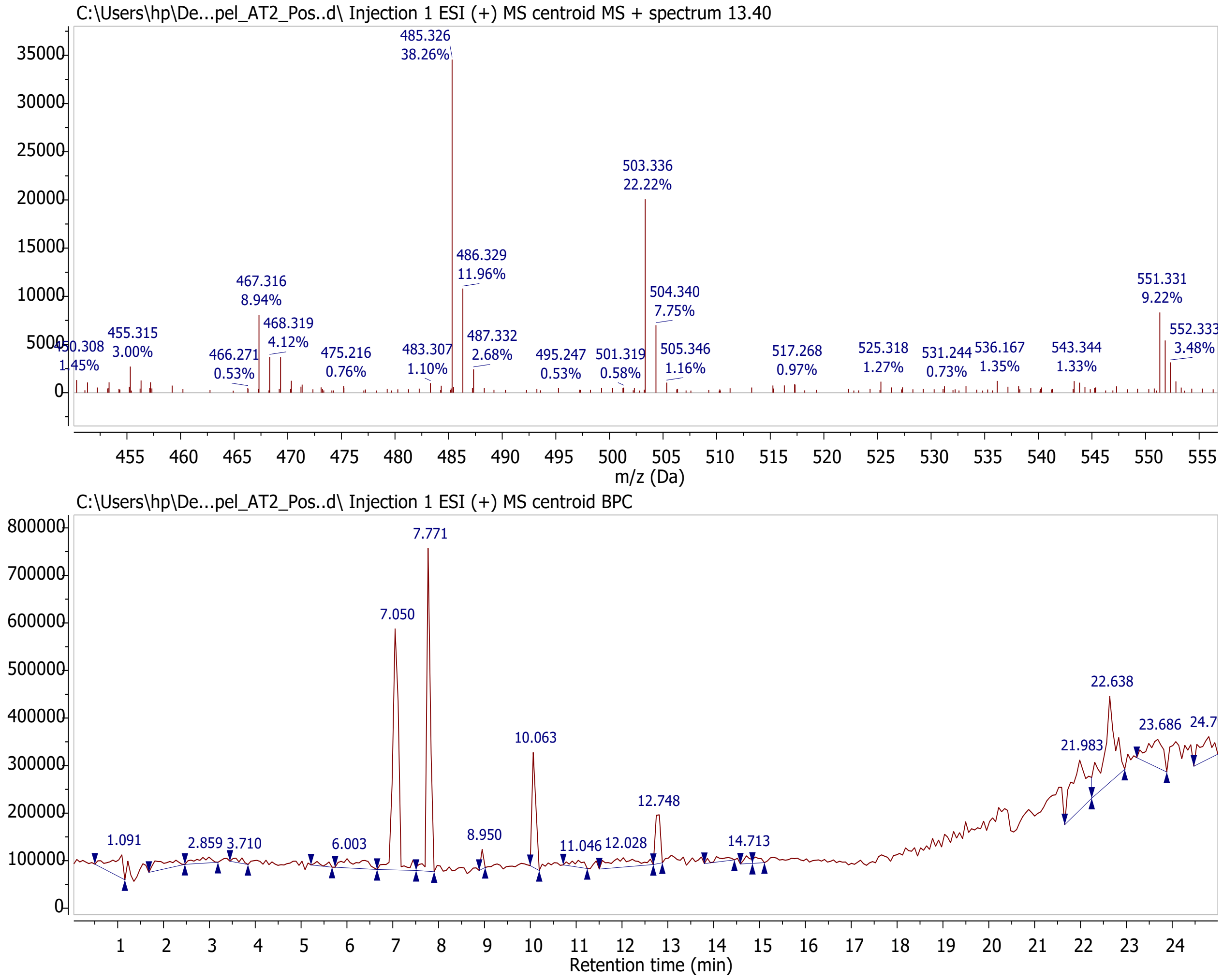

Fig. S15 Mass spectrum of rotundioic acid from *Terminalia chebula* (bark)

This journal is © The Royal Society of Chemistry 20xx

Please do not adjust margins

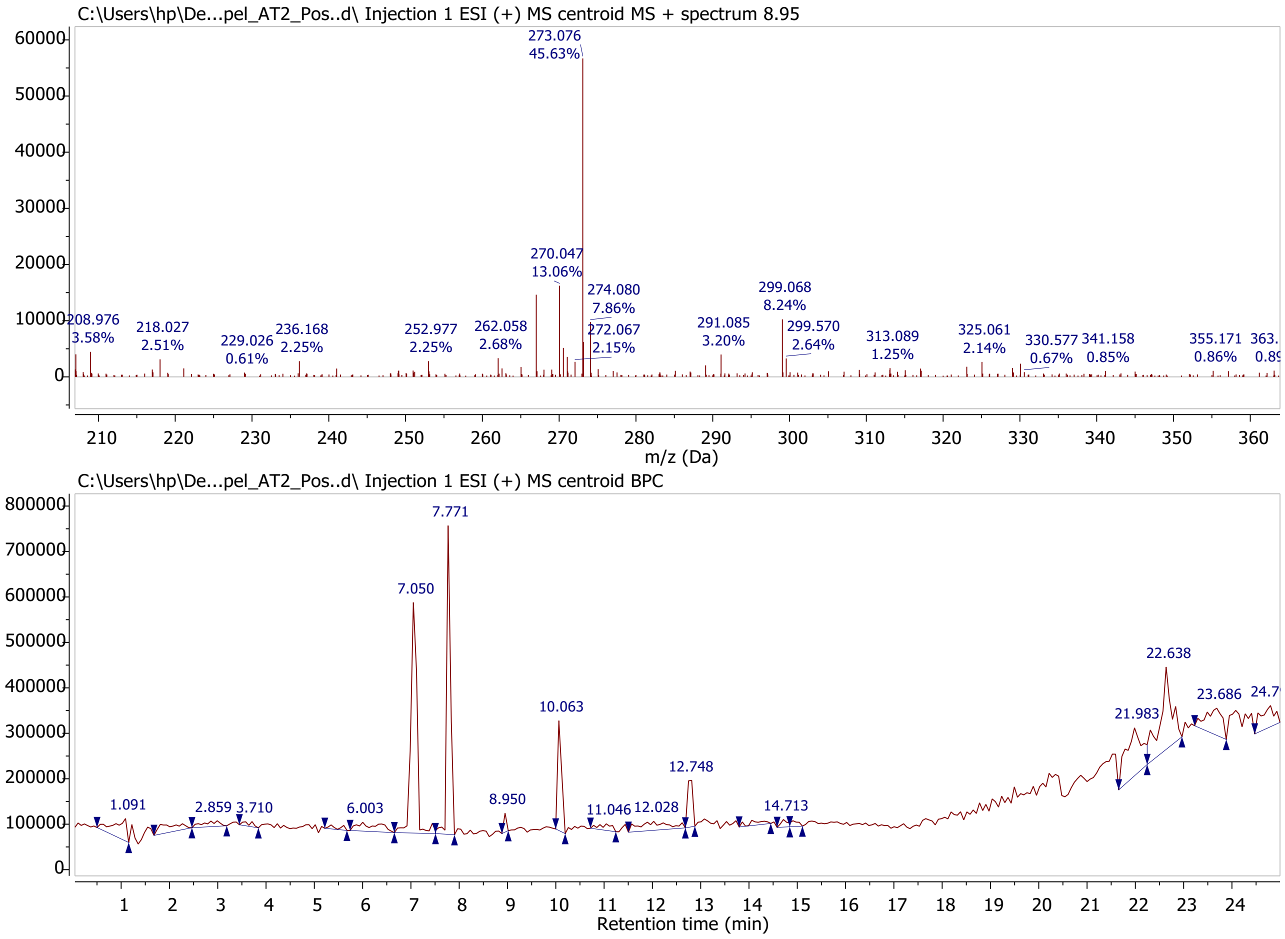

Fig. S16 Mass spectrum of butin from *Terminalia chebula* (bark)

Please do not adjust margins

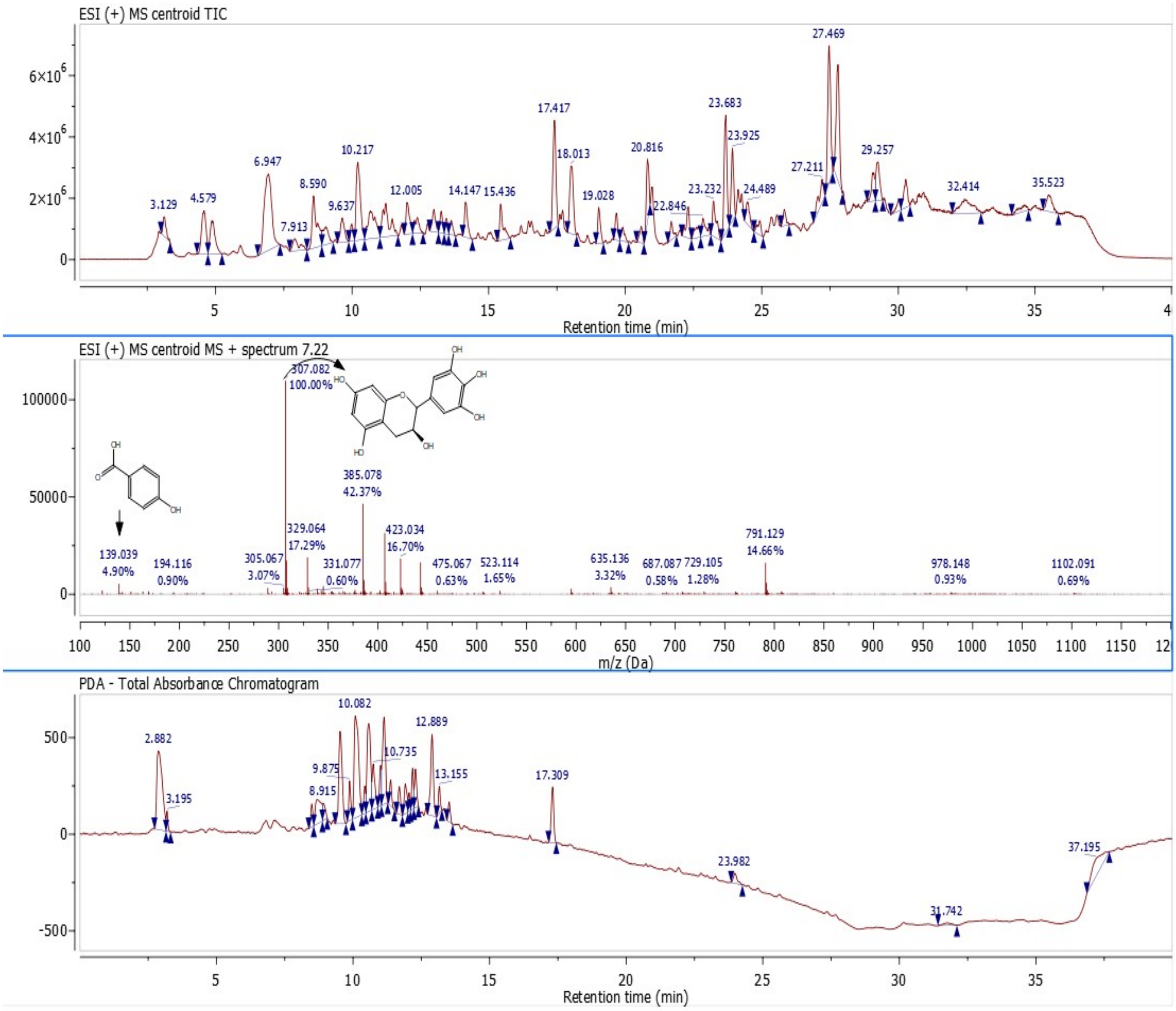

Fig. S17 Mass spectrum of gallocatechin or epigallocatechin from *Cleistocalyx operculatus*

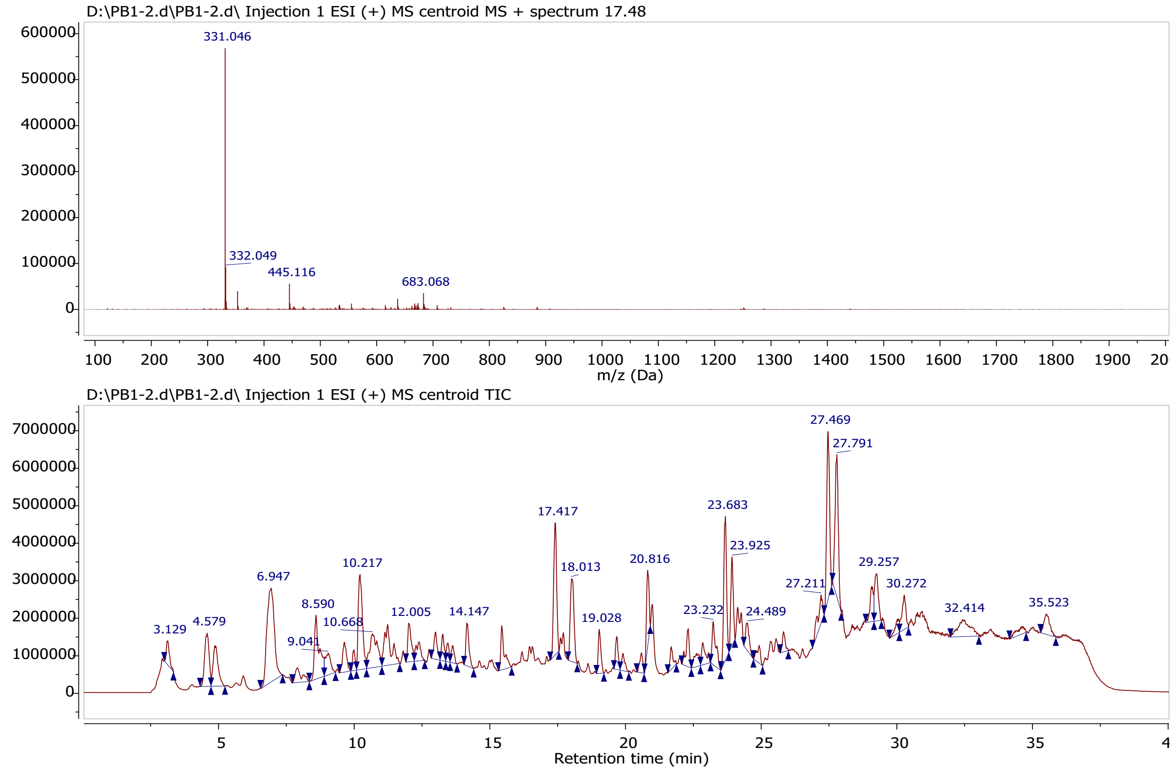

Fig. S18 Mass spectrum of 4,4′-di-*O*-methylellagic acid from *Cleistocalyx operculatus*

Please do not adjust margins

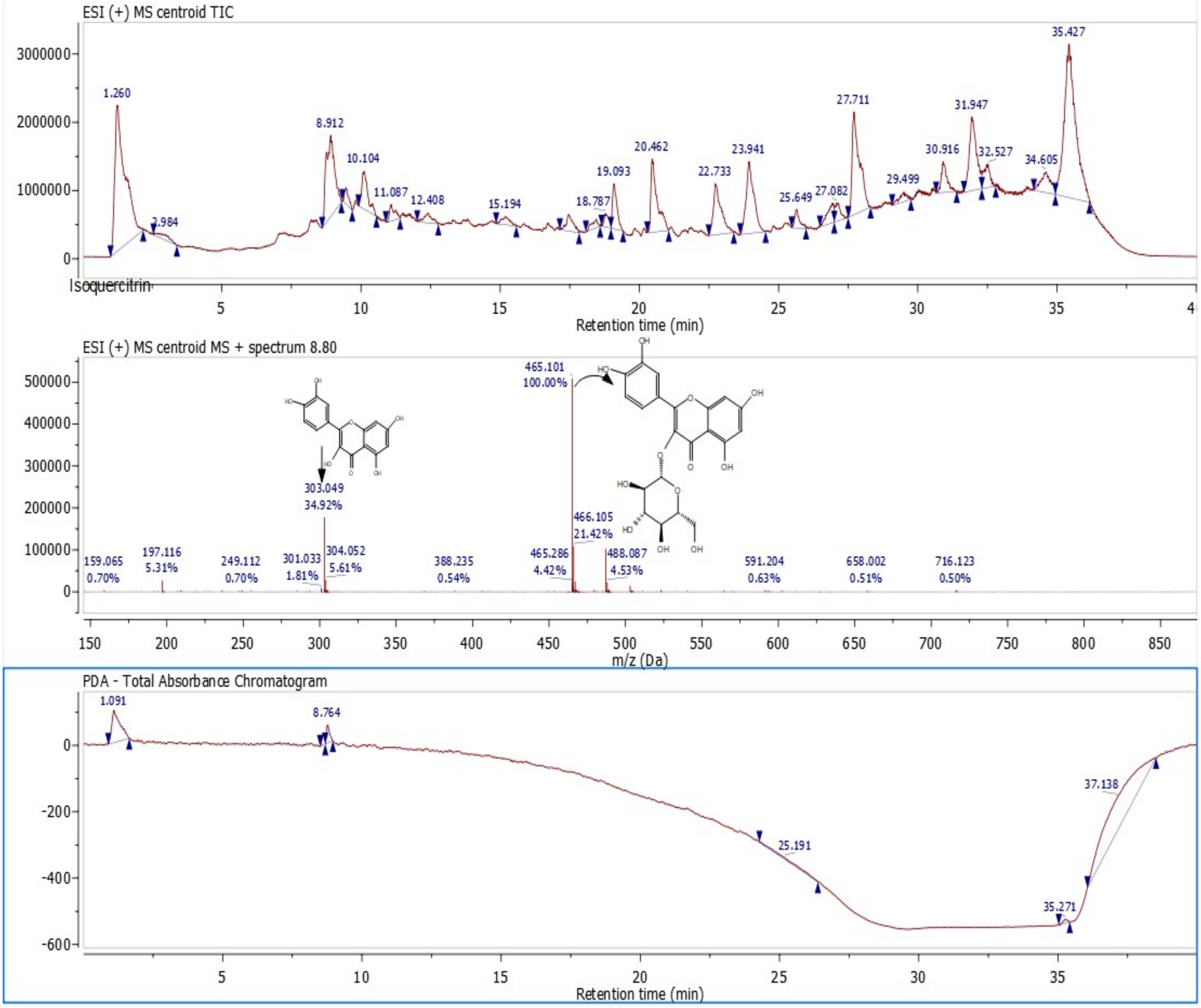

Fig. S19 Mass spectrum of isoquercitrin from *Ficus lacor*

This journal is © The Royal Society of Chemistry 20xx

Please do not adjust margins

Please do not adjust margins

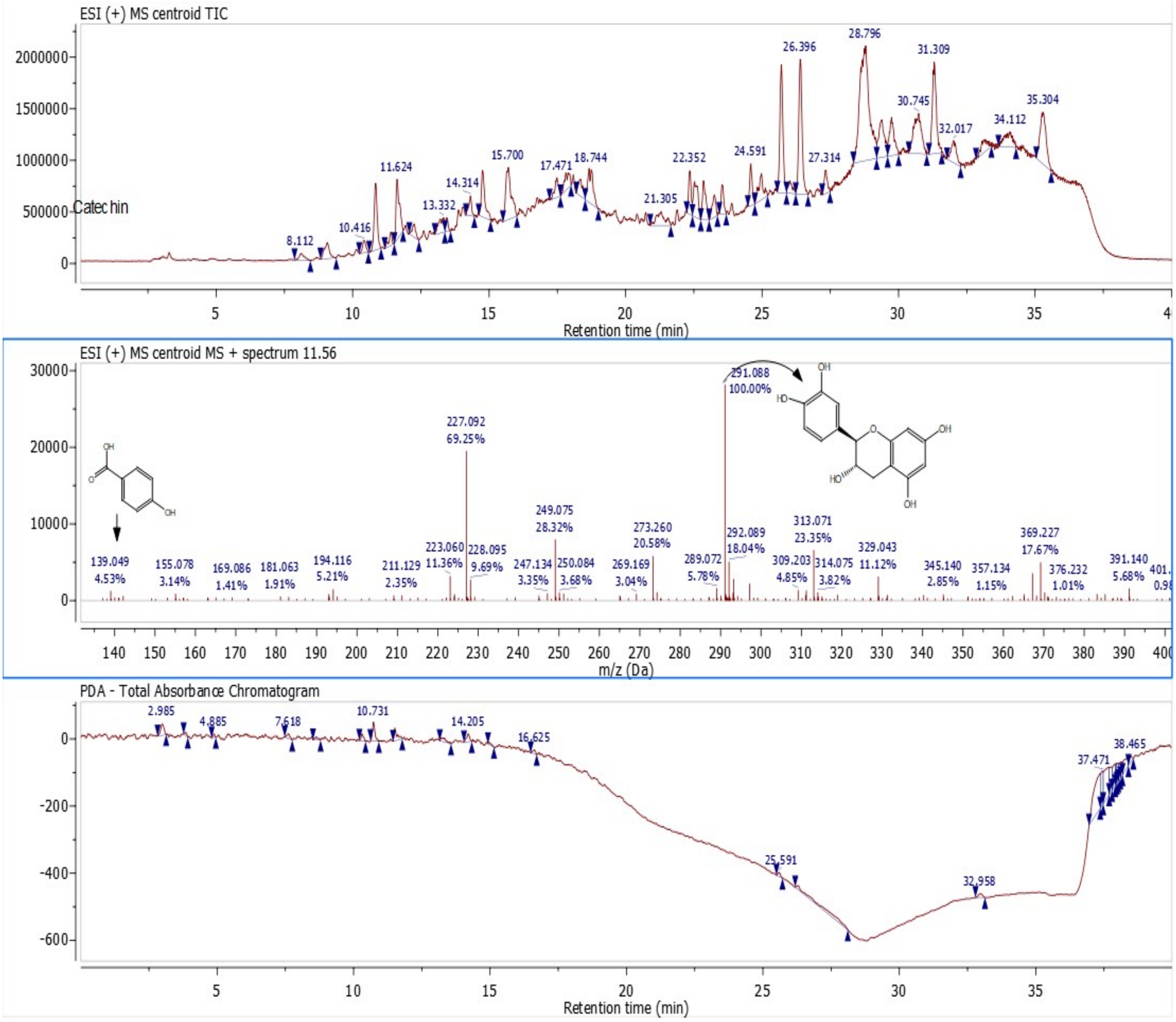

Fig. S20 Mass spectrum of catechin from *Ficus Semicordata*

This journal is © The Royal Society of Chemistry 20xx

Please do not adjust margins

Please do not adjust margins

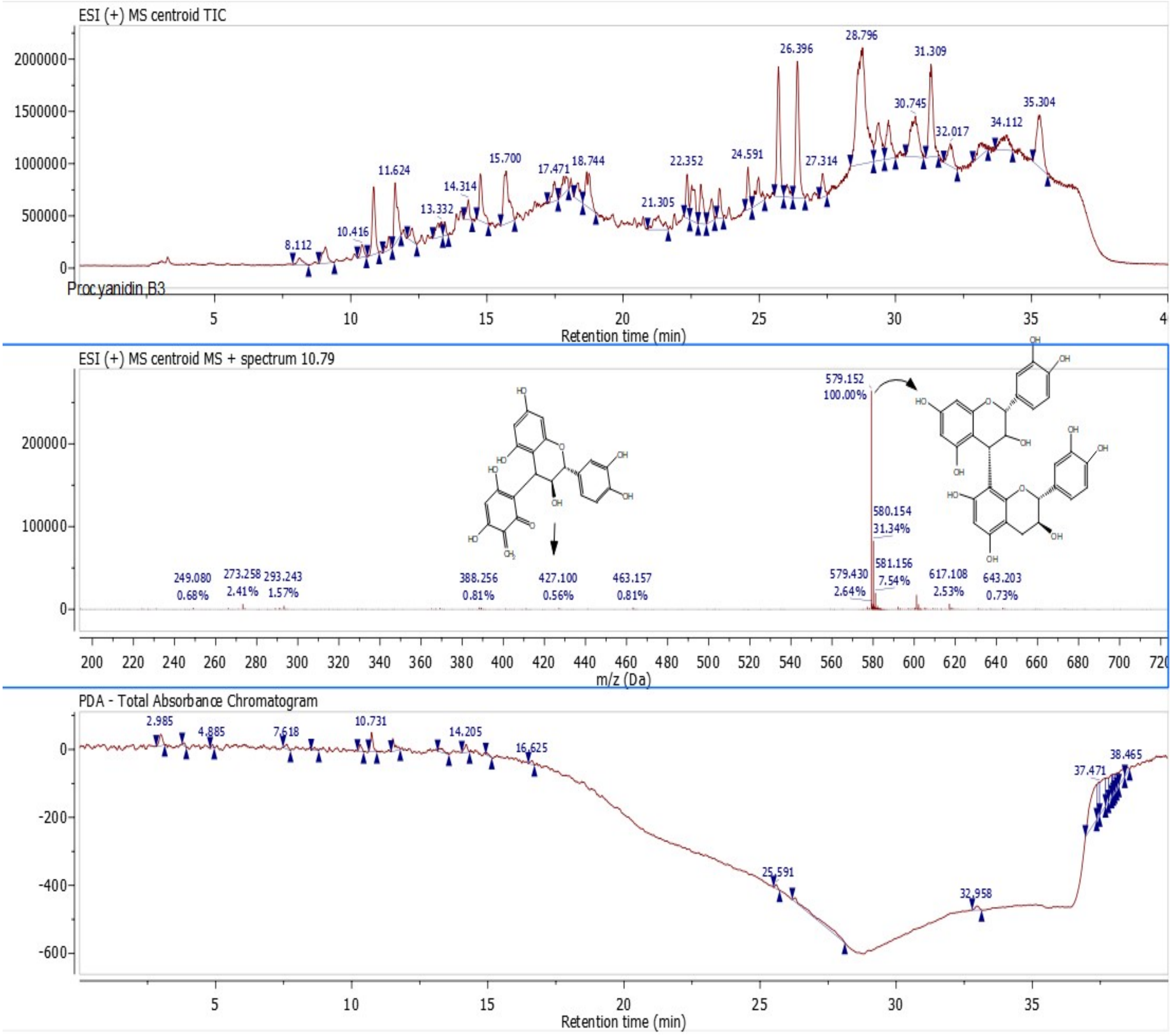

Fig. 21 Mass spectrum of procyanidin B2 from *Ficus semicordata*

This journal is © The Royal Society of Chemistry 20xx

Please do not adjust margins

Please do not adjust margins

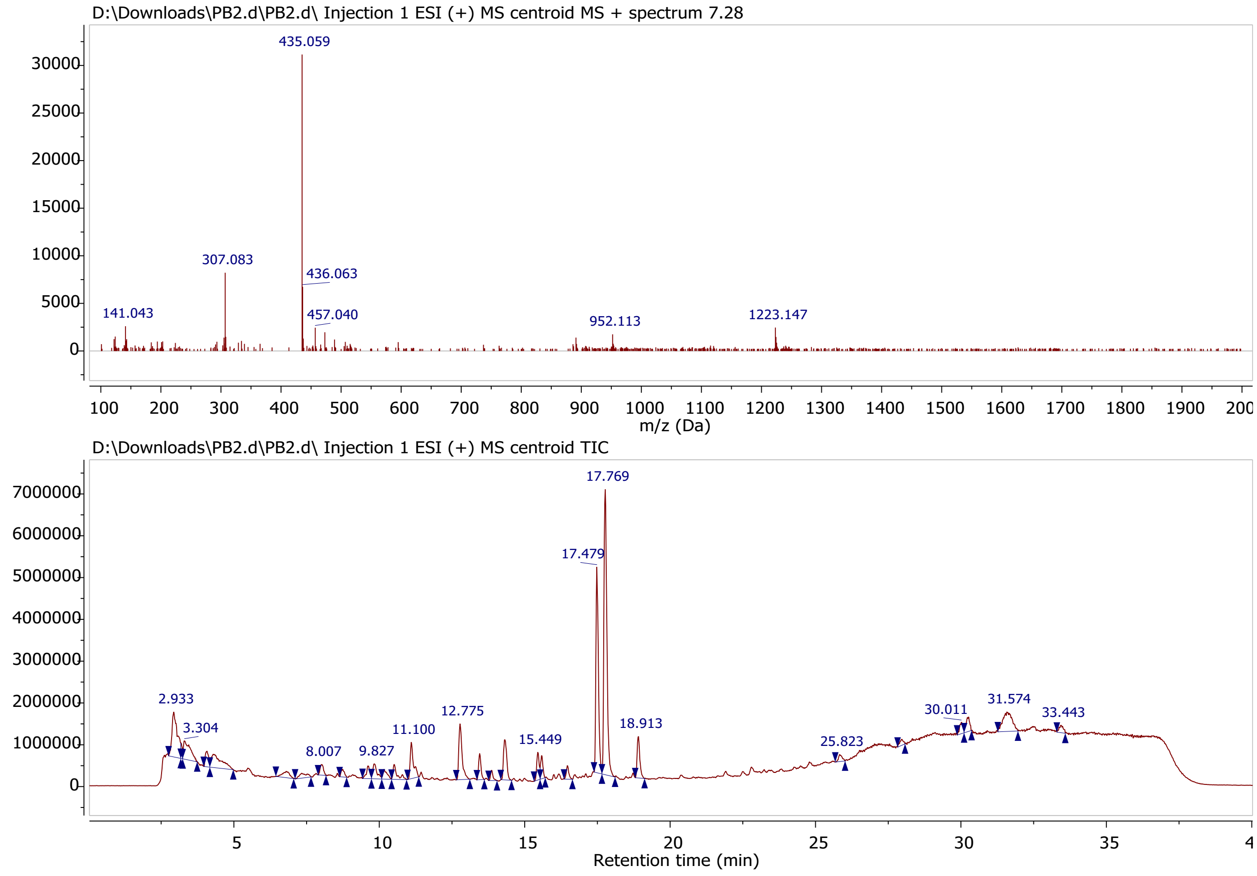

Fig. S22 Mass spectrum of ellagic acid-*O*-pentoside from *Cleistocalyx operculatus*

Please do not adjust margins

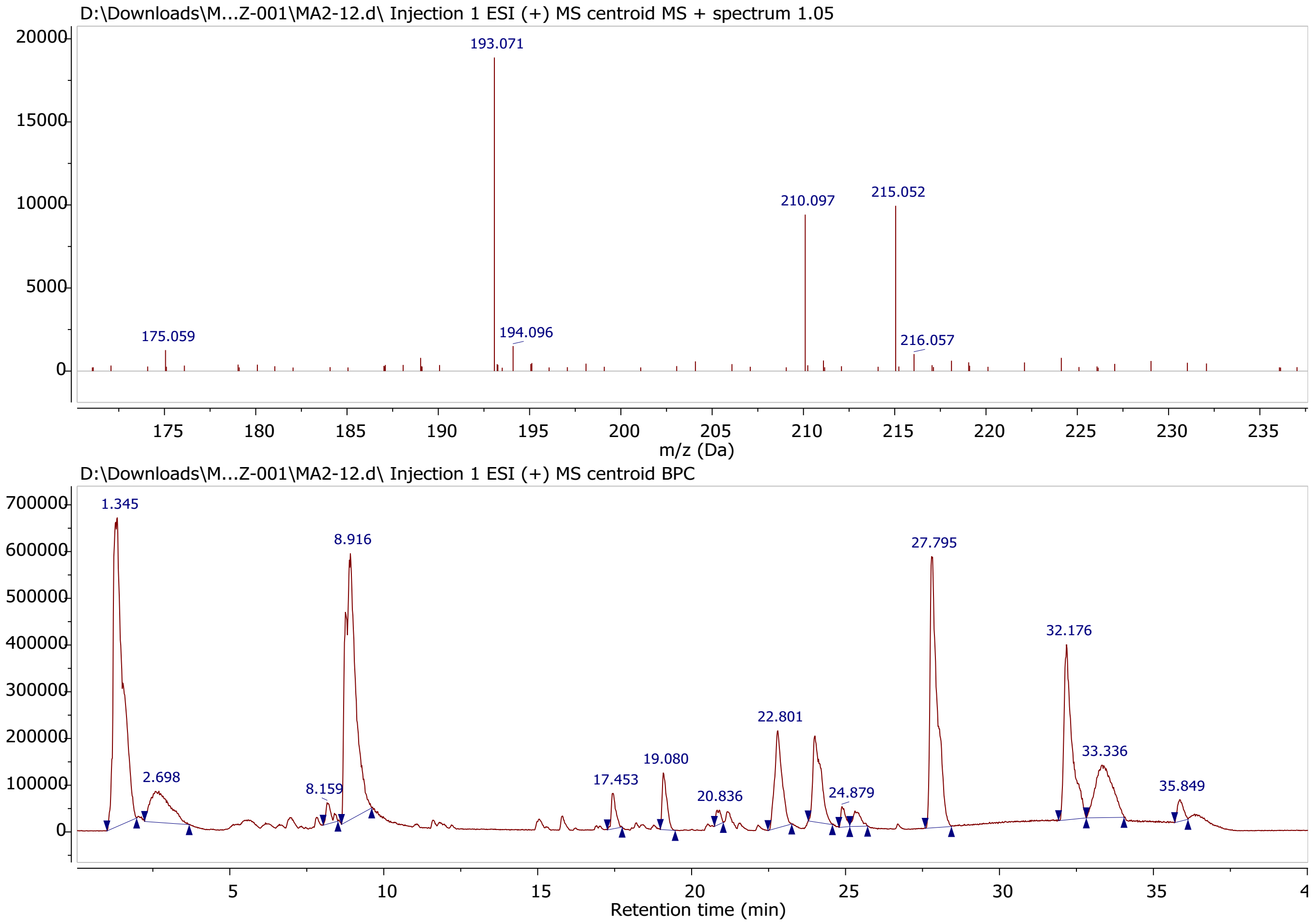

Fig. S23 Mass spectrum of quinic acid from *Ficus lacor*

 This journal is © The Royal Society of Chemistry 20xx

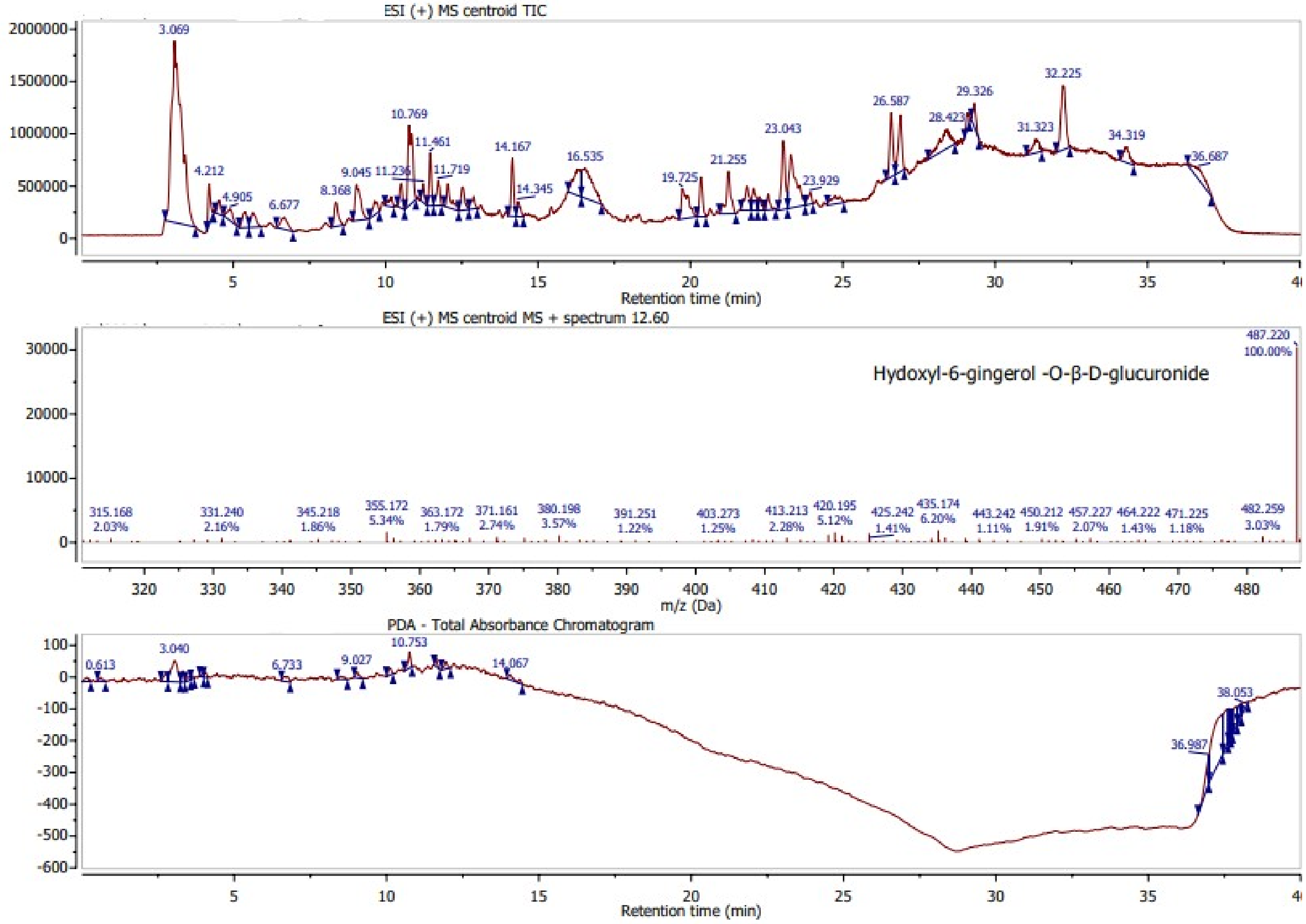

Fig. S24 Mass spectrum of hydoxyl-6-gingerol-*O*-β-D-glucuronide from *Ficus semichordata*

Please do not adjust margins

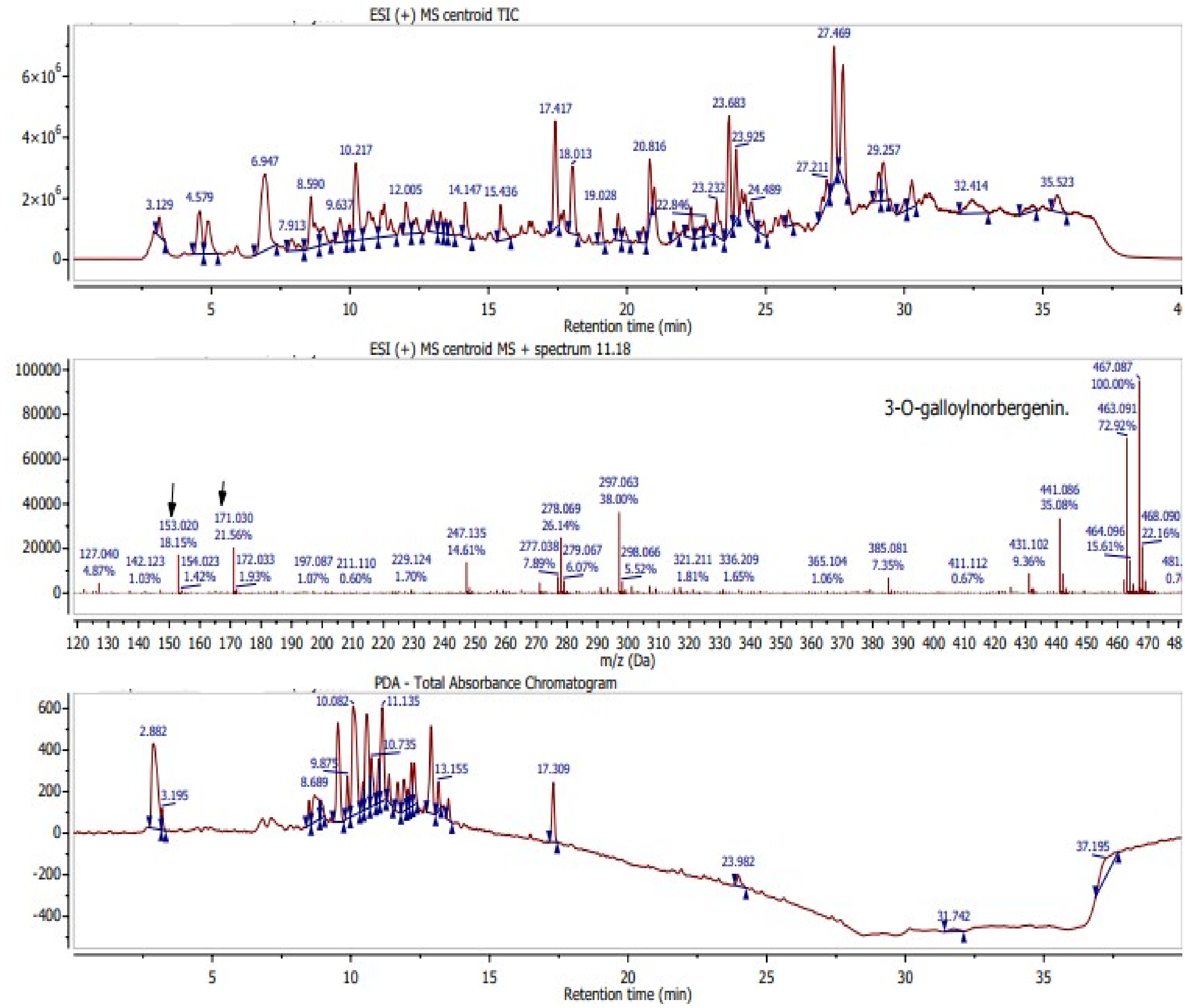

Fig. S25 Mass spectrum of 3-*O*-galloylnorbergenin from *Cleistocalyx operculatus*

 This journal is © The Royal Society of Chemistry 20xx

Please do not adjust margins

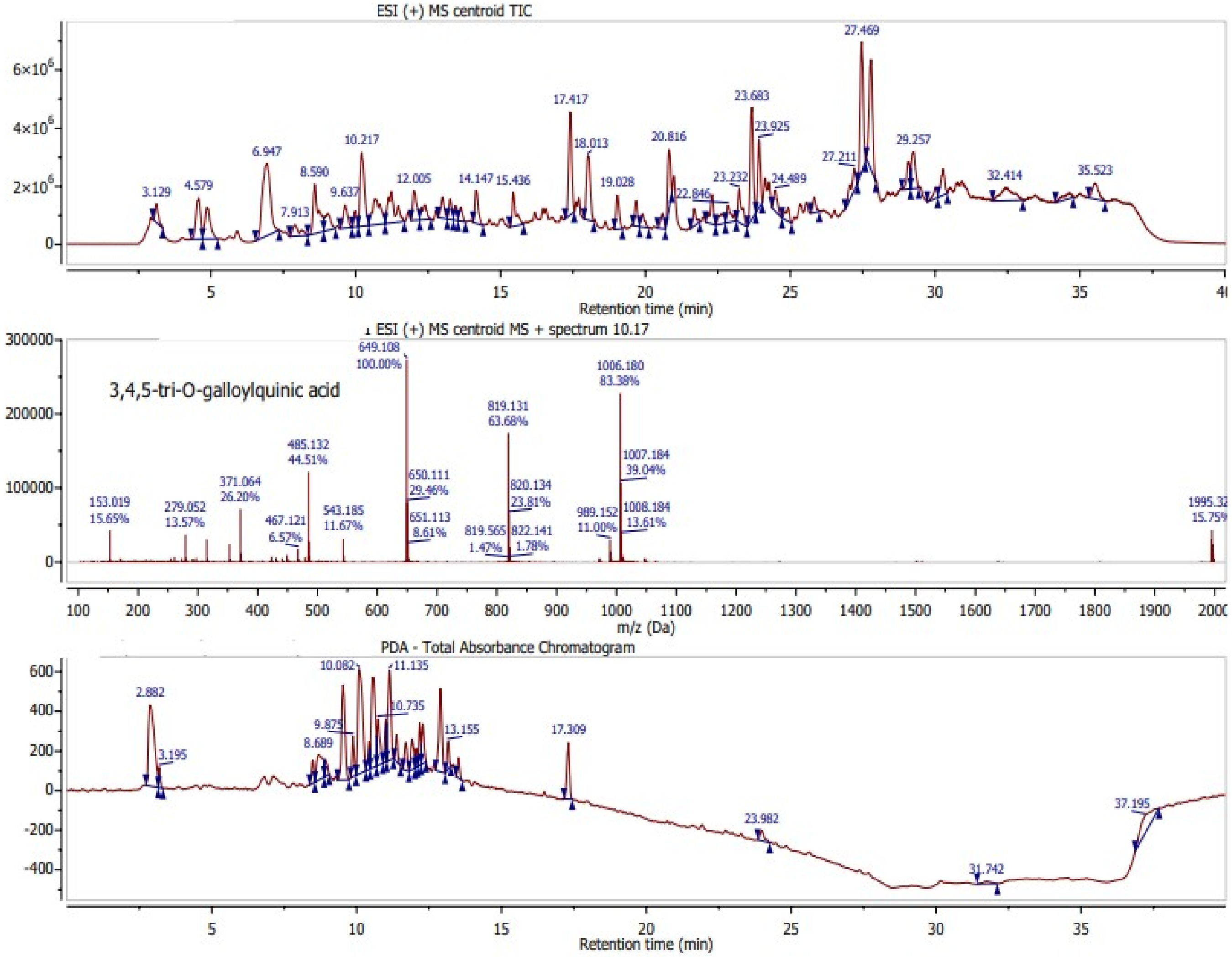

Fig. S26 Mass spectrum of 3,4,5-tri-*O*-galloylquinic acid from *Cleistocalyx operculatus*

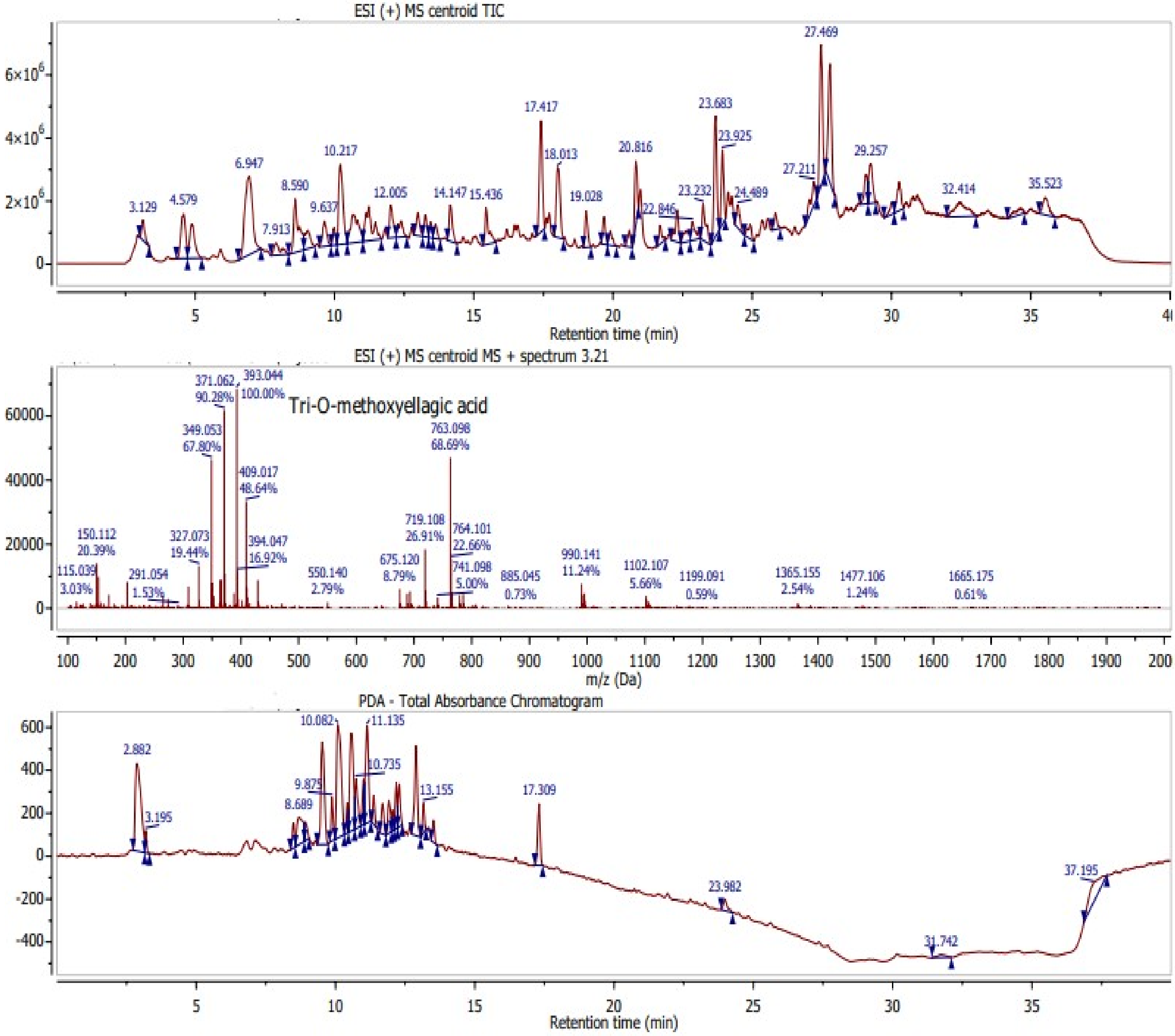

Fig. S27 Mass spectrum of tri-*O*-methoxyellagic acid from *Cleistocalyx operculatus*

This journal is © The Royal Society of Chemistry 20xx

Please do not adjust margins

Please do not adjust margins

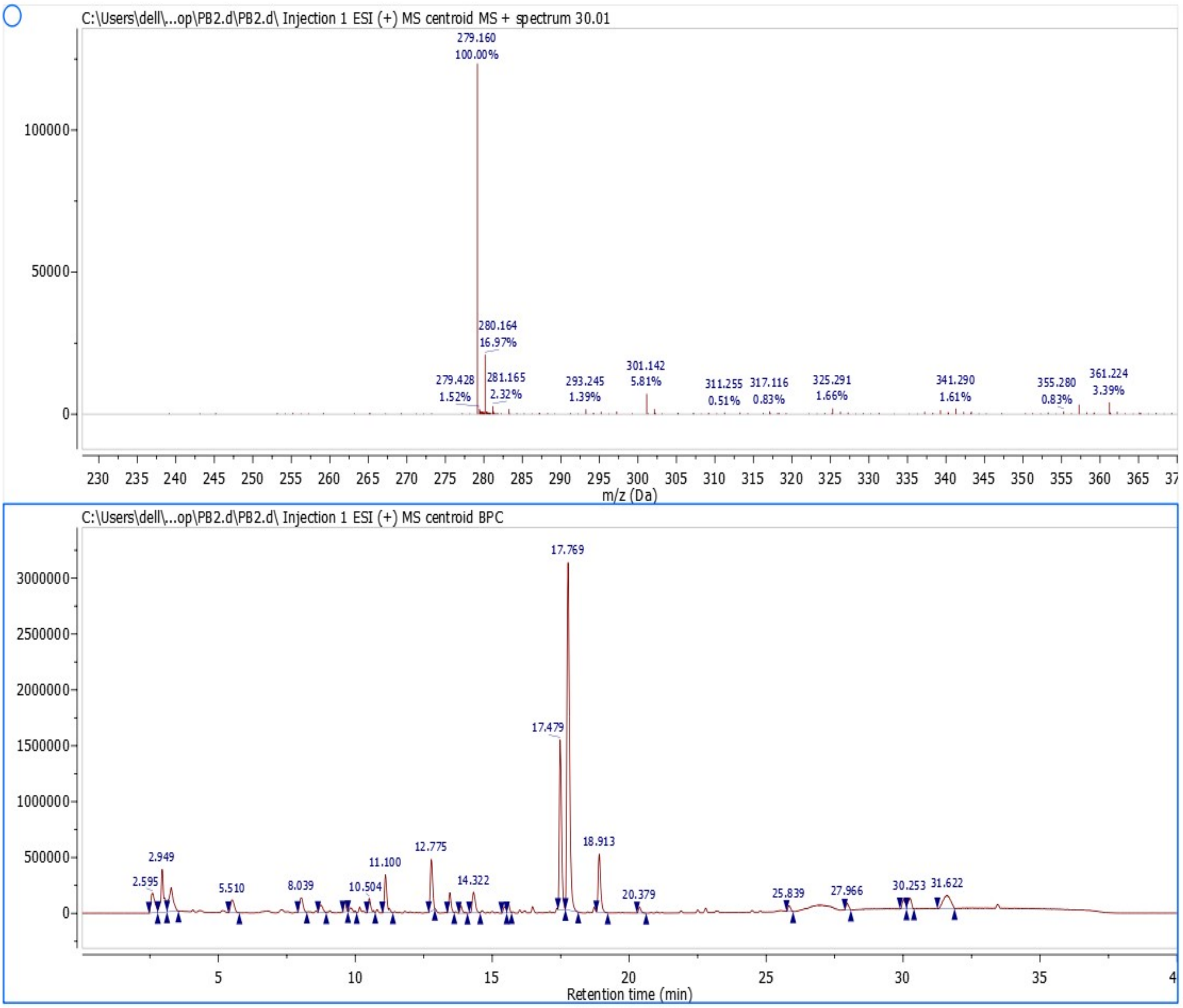

Fig. S28 Mass spectrum of butyl isobutyl phthalate from *Cleistocalyx operculatus*

Please do not adjust margins

Please do not adjust margins

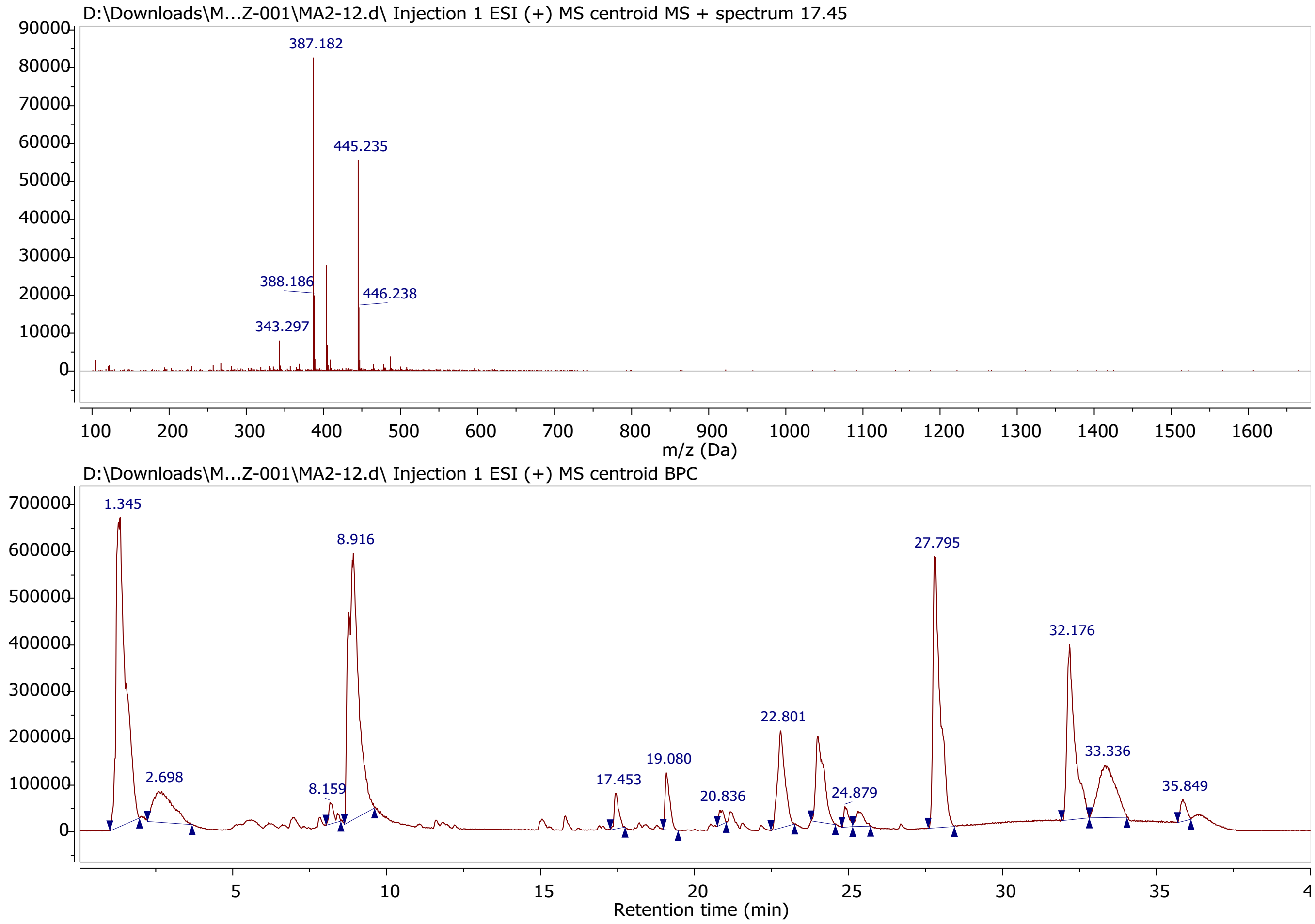

Fig. S29 Mass spectrum of eudesmin from *Ficus lacor*

 This journal is © The Royal Society of Chemistry 20xx

Please do not adjust margins

Please do not adjust margins

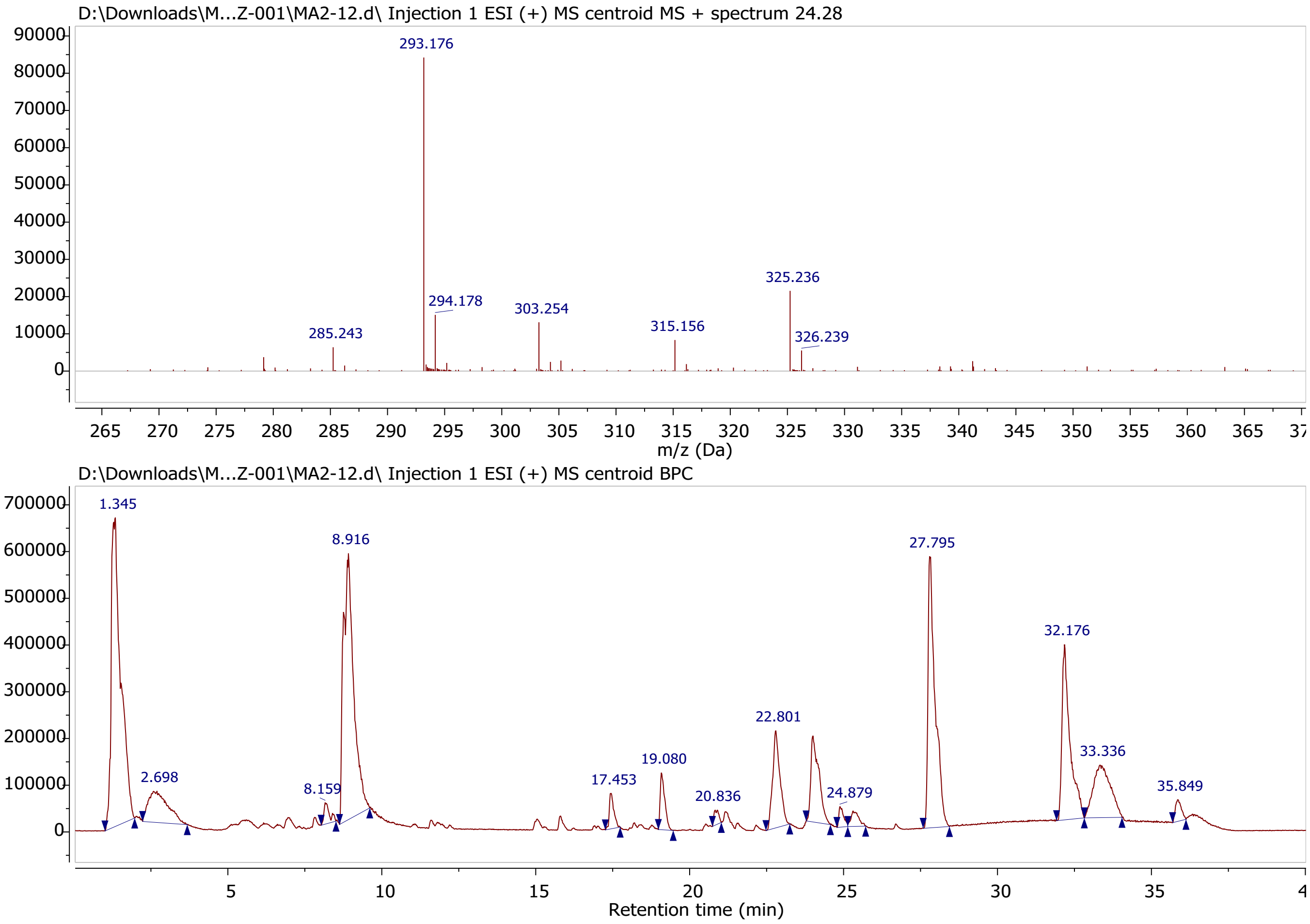

Fig. S30 Mass spectrum of lasiodiplodin from *Ficus lacor*

Please do not adjust margins

Please do not adjust margins

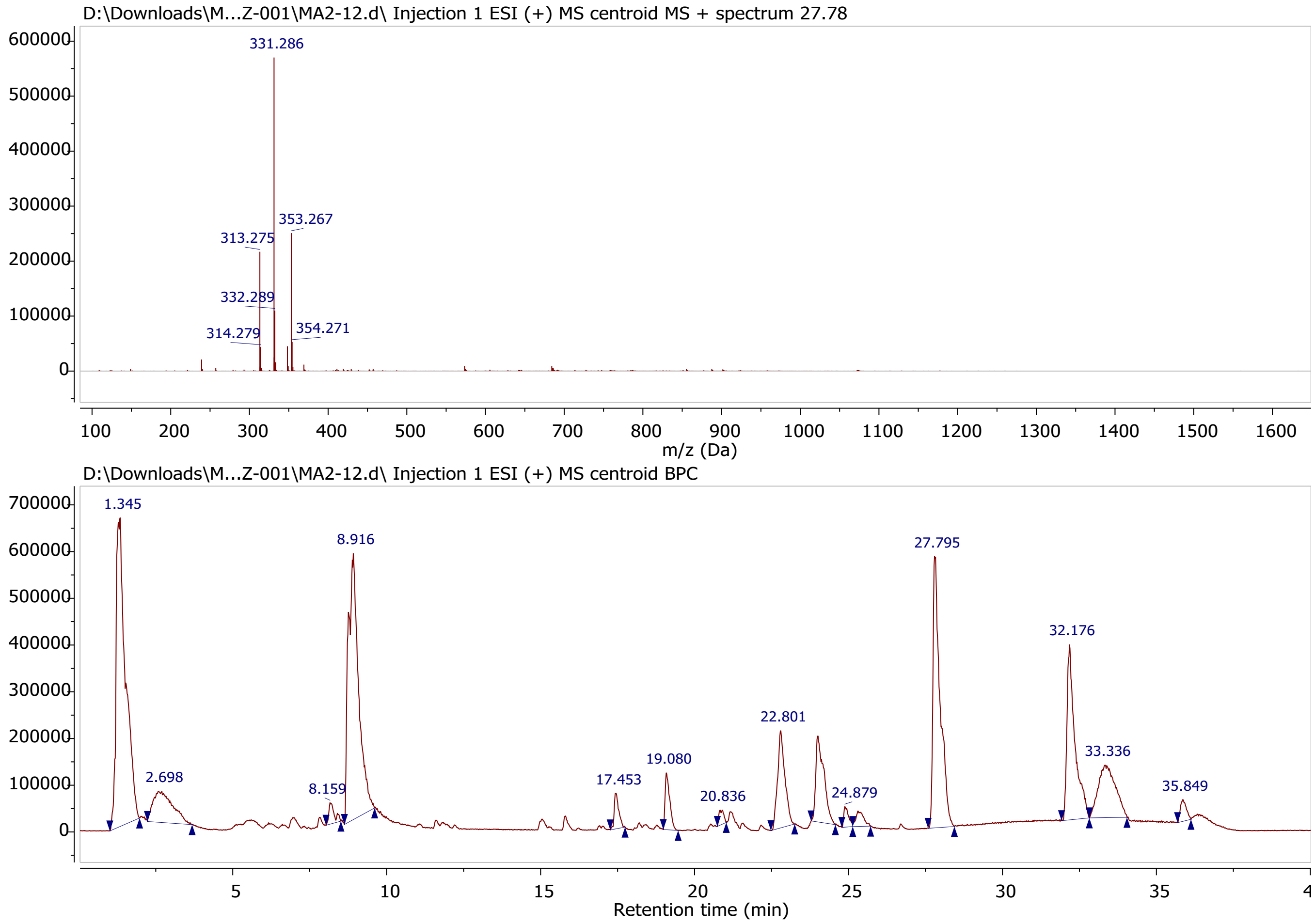

Fig. S31 Mass spectrum of 1-monopalmitin from *Ficus lacor*

This journal is © The Royal Society of Chemistry 20xx

Please do not adjust margins

Please do not adjust margins

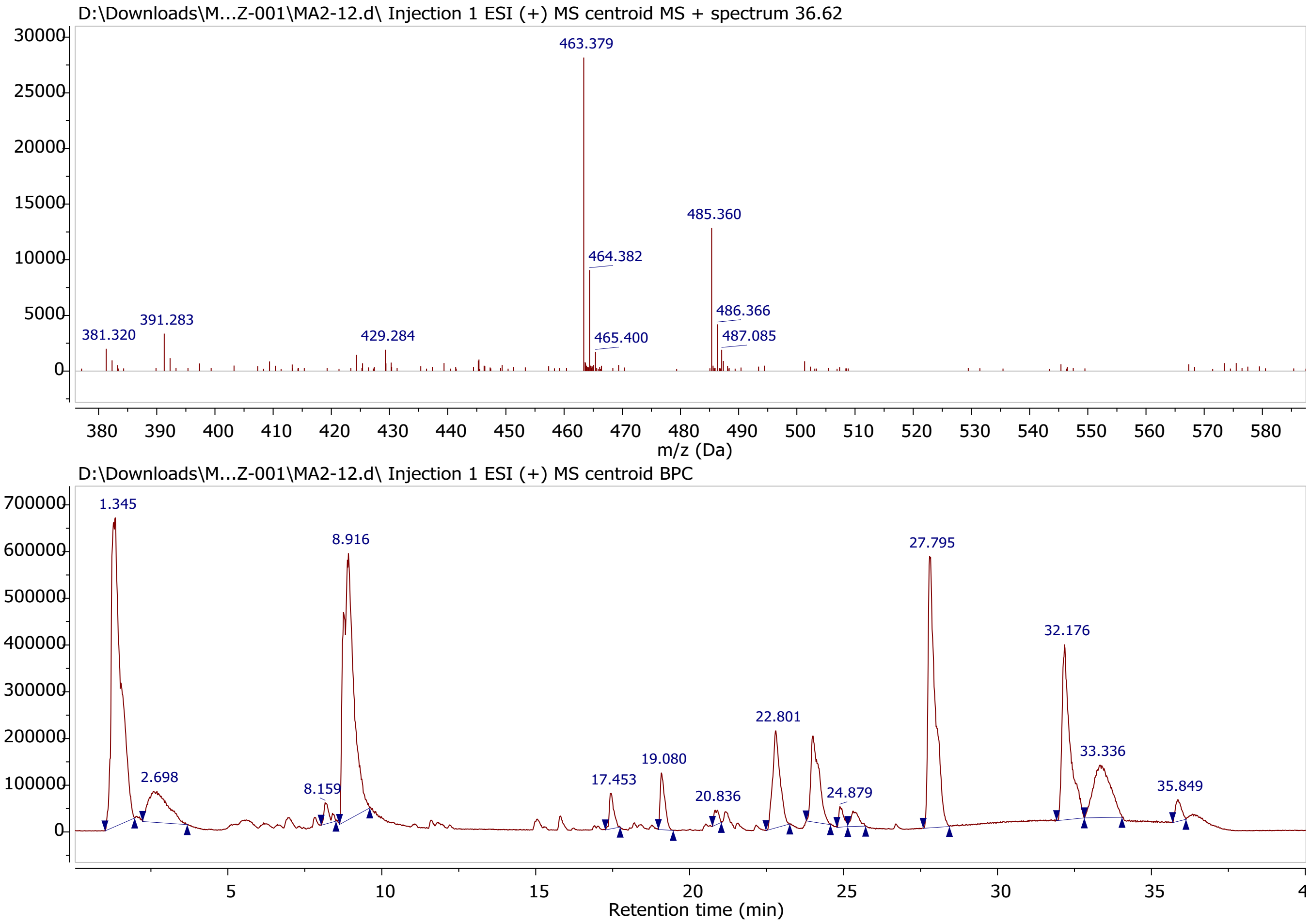

Fig. S32 Mass spectrum of α-tocospiro B from *Ficus lacor*

Please do not adjust margins
